# Bioactivity of Eugenol: A Potential Antibiotic Adjuvant with Minimal Ecotoxicological Impact

**DOI:** 10.3390/ijms25137069

**Published:** 2024-06-27

**Authors:** Natalia Ferrando, María Rosa Pino-Otín, Eva Terrado, Diego Ballestero, Elisa Langa

**Affiliations:** 1 Facultad de Ciencias de la Salud, Universidad San Jorge, Campus Universitario, Autovía Mudéjar, km. 299, 50830 Villanueva de Gállego, Spain; nferrando@usj.es (N.F.); rpino@usj.es (M.R.P.-O.); dballestero@usj.es (D.B.); 2 Facultad de Educación, Universidad de Zaragoza, Pedro Cerbuna 12, 50009 Zaragoza, Spain; eterrado@unizar.es

**Keywords:** eugenol, antimicrobial synergy, antibiotic adjuvant, acute ecotoxicity, non-target organisms, environmental impact

## Abstract

Combining commercial antibiotics with adjuvants to lower their minimum inhibitory concentration (MIC) is vital in combating antimicrobial resistance. Evaluating the ecotoxicity of such compounds is crucial due to environmental and health risks. Here, eugenol was assessed as an adjuvant for 7 commercial antibiotics against 14 pathogenic bacteria in vitro, also examining its acute ecotoxicity on various soil and water organisms (microbiota, *Vibrio fischeri*, *Daphnia magna*, *Eisenia foetida*, and *Allium cepa)*. Using microdilution methods, checkerboard assays, and kinetic studies, the MICs for eugenol were determined together with the nature of its combinations with antibiotics against bacteria, some unexposed to eugenol previously. The lethal dose for the non-target organisms was also determined, as well as the Average Well Color Development and the Community-Level Physiological Profiling for soil and water microbiota. Our findings indicate that eugenol significantly reduces MICs by 75 to 98%, which means that it could be a potent adjuvant. Ecotoxicological assessments showed eugenol to be less harmful to water and soil microbiota compared to studied antibiotics. While *Vibrio fischeri* and *Daphnia magna* were susceptible, *Allium cepa* and *Eisenia foetida* were minimally affected. Given that only 0.1% of eugenol is excreted by humans without metabolism, its environmental risk when used with antibiotics appears minimal.

## 1. Introduction

Historically, natural products and their analogues have played a significant role in pharmacotherapy, particularly in treating infectious diseases [1]. These products possess diverse active components, enabling them to target various bacterial strains, thus facilitating combinational therapy through multiple modes of action [2]. Terpenes, derived from the mevalonate metabolic pathway, constitute a significant natural product family with diverse activities that have been extensively researched [3,4]. They can kill or hinder microorganism growth depending on their concentration, despite their low water solubility [5,6]. Monoterpenes’ antimicrobial efficiency varies based on their solubility and hydrogen bond ability, but it is not significantly impacted by the presence of double bonds or cyclic moieties in their structure [5]. Aromatic compounds with alcohol groups, like eugenol (EUG), have strong inhibitory activity against microorganisms [5].

EUG, classified as a phenylpropanoid, is a derivative of allyl chain-substituted guaiacol and it is biosynthesized by plants from phenylalanine [7,8,9]. EUG exhibits weak acidic properties and slight solubility in water, while being readily soluble in organic solvents [10,11,12]. It is typically derived from the natural essential oils found in plants belonging to the *Lamiaceae*, *Lauraceae*, *Myrtaceae*, and *Myristicaceae* families. EUG is notably abundant in clove oil (*Syzygium aromaticum*), serving as its principal constituent [13,14,15], but it can be synthetically manufactured too [14,15,16]. This terpenoid has exhibited a range of beneficial properties, including antimicrobial activity against numerous human pathogens, encompassing a broad spectrum of Gram-positive and Gram-negative bacteria, fungi, and various parasites [13,14,16,17,18,19,20,21]. Its potential as a new antibiotic against resistant bacteria is attributed to its natural origin and multifaceted mechanisms of action [22]. Harnessing secondary plant metabolites, like EUG, as antimicrobial and/or adjuvant presents a cost-effective and innovative approach to developing novel strategies against antimicrobial resistance (AMR). Notably, very few instances of plant-based chemical resistance have been documented to date [23,24].

A key approach to combatting AMR involves combining commercial antibiotics (ABXs) with substances such as adjuvants, some which are natural products, like monoterpenoids, to reduce the minimum inhibitory concentration (MIC) of the former. This established strategy is effective against resilient pathogens [25,26,27,28]. When two substances interact, they can exhibit synergistic, additive, or antagonistic effects [29]. Synergy occurs when the combined effect of two compounds surpasses the sum of their individual effects, with a lower dosage and toxicity, while antagonism is when a substance hinders the effect of another. On the other hand, addition happens when the effect of the mixture equals the adding of the individual effects. Synergy is the expected behavior when combining a commercial ABX and an adjuvant, particularly against resistant microorganisms [30]. EUG was identified as Generally Recognized As Safe (GRAS) by prominent regulatory bodies such as the Food and Drug Administration (FDA) [31], the World Health Organization (WHO), and the Council of Europe [32]. This recognition would underscore its safety for both human health and the environment [33], but might jeopardize future economical investment as a plausible adjuvant. Moreover, its safety profile has facilitated its application in pharmaceutical preparations and as a flavoring agent, stabilizer, antioxidant, antiseptic, and anesthetic in dental procedures [34].

Analyzing soil and water ecotoxicity of new antimicrobials and their adjuvants is vital due to their significant environmental and health risks. Research highlights the role of environmental pollutants, including antimicrobials, in spreading AMR [35]. By-products resulting from the modification or the degradation of antimicrobials have been identified in various environmental sources, emphasizing the need for monitoring [36]. Antimicrobials in the environment could be toxic for algae and plants and inhibit microbial growth, affecting microbial diversity and thus negatively impacting soil enzymatic and metabolic activities [37,38]. Therefore, assessing the ecotoxicity of new antimicrobials in soil and water is essential to understand their effects on ecosystems, microbial communities, and human health. Such analyses enable the risk assessment, mitigation strategy development, and protection of environmental and human well-being. Assessments of EUG’s antimicrobial and/or adjuvant activity should be accompanied by ecotoxicity studies, which are very scarce, addressing potential adverse effects on aquatic and terrestrial ecosystems and human and animal health, in line with the One Health strategy promoted by the WHO [39].

Given these considerations, the objective of this research was doubled:

(a) To assess the antimicrobial activity of EUG alone and its ability to enhance the bacterial inhibitory effect of commercial ABXs against clinically relevant Gram-positive and Gram-negative bacteria to find the synergistic combinations (in which the EUG would be the adjuvant) lowering MIC and thus allowing the reduction of ABXs’ doses.
(b) To quantify the environmental impact of EUG by employing terrestrial and aquatic individual indicators alongside genetically sequenced microbial communities sourced from soil and river environments to obtain a more realistic environmental perspective of its potential use as an ABX adjuvant.

By examining both the antimicrobial efficacy and environmental impact of EUG, this research provides new insights into its dual potential, enhancing antibiotic effectiveness while ensuring ecological safety.

## 2. Results and Discussion

### 2.1. Assessment of Eugenol Antimicrobial Activity

EUG was tested against 14 Gram-positive (*Bacillus subtilis*, *Listeria monocytogenes*, *Streptococcus agalactiae*, and *Staphylococcus aureus*) and Gram-negative (*Acinetobacter baumannii*, *Enterococcus faecalis*, *Escherichia coli*, *Klebsiella aerogenes*, *Klebsiella pneumoniae*, *Pasteurella aerogenes*, *Pseudomonas aeruginosa*, *Salmonella enterica,* and *Serratia marcescens*) bacterial strains. Their MIC results are given in Table 1. EUG could not be tested against *P. mirabilis* since the DMSO concentration required to dissolve it was toxic for these bacteria, whereas it was not for the other bacteria [2], as mentioned in Section 3.3.

Our study, that reports microdilution MIC values for the first time for Gram-positive bacteria such as *L. monocytogenes*, *S. agalactiae,* and *S. aureus*, established MICs of 1000 μg/mL (see Table 1) for these strains. We compared our MIC values with those observed for different strains of the same bacteria using different methods. For example, the MIC for non-resistant strains of *S. aureus* determined by Walsh et al. [45] varied from 106 to 1590 μg/mL, while those observed by Gallucci et al. [5] and Hammer and Heel [46] were approximately 33,430 μg/mL and from 127.2 to 8480 μg/mL, respectively. Perugini Biasi-Garbin et al. [47] analyzed the MIC of several strains of *S. agalactiae* and found values ranging from 1325 to 5300 μg/mL, which are close to ours. The reported MIC values for *L. monocytogenes* varied from 67 to 1024 μg/mL according to different authors [48,49,50,51,52,53], some of which are also close to our values. For *B. subtilis*, no reported values for the isolated compound were found.

Previous research suggested that EUG disrupts bacterial membranes, increasing their permeability [46,47,48,54,55,56]. Additionally, EUG might inhibit ATPase. This could alter the efflux pump activity, especially in *L. monocytogenes* and *S. agalactiae* [48,57], resulting in the higher intracellular concentration of the ABX. Both in vivo and in vitro studies demonstrate reduced *L. monocytogenes* virulence upon EUG treatment [48,58,59,60,61,62], highlighting its potential as an adjuvant to conventional antibiotics, against *L. monocytogenes* and possibly other pathogens. Interestingly, *L. monocytogenes* strains did not develop resistance to EUG after exposure to sub-inhibitory concentrations [53].

Concerning Gram-negative bacteria, reported MIC values for the same strains as ours using the macro- or microdilution methods were found for *E. coli*, *P. aeruginosa*, *S. enterica,* and *S. marcescens* (Table 1). In *E. coli*, the MIC values exhibit considerable variations not only with our results, but also in the studies cited in Table 1. However, for *S. enterica* and *S. marcescens,* they are closer to our MIC values. In the case of *P. aeruginosa,* no comparison can be made because the value mentioned by the authors is not precisely determined and above 1000 [42] or 2000 μg/mL [40]. For the remaining bacteria, no values for the same strains were found with the macro- or microdilution methods, so this comparison was performed for different strains. *A. baumannii*’s MIC ranged from 90.5 to 304 μg/mL [63] and 318 μg/mL [64], slightly lower than ours. *E. faecalis*’ MIC was 1060 μg/mL in the study conducted by Hammer and Heel [46], half of our value. Lastly, no EUG MIC was found for *P. aerogenes* and *K. aerogenes*. Our study was thus the first one to provide information about the inhibitory effect of EUG on these species.

The variation of MIC values of Gram-negative bacteria is probably due to differences in the complexity of their outer membrane. Within the group of Gram-negative bacteria, the lowest MIC values were obtained for *A. baumannii*, *K. aerogenes*, *K. pneumoniae*, and *P. aerogenes* (500 μg/mL, Table 1). These low values suggest a potential specificity of EUG against these bacteria. In the case of *A. baumannii*, consistently with the studies of Karumathil et al. [64], this could be attributed mainly to an inhibition of the AdeABC efflux pump. In *K. pneumoniae*, EUG was shown to induce membranous lipid peroxidation resulting from the production of ROS [65,66,67], leading to membrane disruption. ROS production caused by EUG might be specific to this bacterium, leading to lower MIC values. Except for *P. mirabilis*, for which experiments were not feasible (see Section 3.3 for more information), the MIC values for the other Gram-negative bacteria exceeded 1000 μg/mL (Table 1). Regarding *E. faecalis*, Hammer and Heel [46] observed significant membrane disruption, while Morgaan et al. [68] attributed EUG’s mode of action on *E. coli* to its lipophilic nature, causing bacterial membrane lipid disintegration, leading to energy depletion and cellular component leakage. Additionally, ATPase inhibition was noted in *E. coli* exposed to EUG [47,69]. For *P. aeruginosa*, membrane disruption was also evident [42,45], although some studies suggested that EUG’s antimicrobial activity might prolong the lag phase of bacterial growth at sub-inhibitory concentrations, possibly due to interactions with primary non-vital bacterial targets [70]. Aljuwayd et al. [71] and Zhao et al. [72] investigated the effects of EUG on *S. Typhimurium* (*S. enterica*) in vivo, observing the destruction of fimbriae, and reductions in adhesion molecules, and virulence factors. Aljuwayd et al. observed decreased pathogenicity and cell counts in chickens with EUG pretreatment, while Zhao et al. reported similar findings and noted EUG’s ability to maintain its intestinal barrier function and Tight Junctions (TJs) protein stability in mice. No specific information regarding the mode of action was available for *S. marcescens* and *P. mirabilis*.

### 2.2. Eugenol Combined with Commercial Antibiotics

The checkboard assay (Table 2) revealed promising interactions between EUG and the conventional ABXs ampicillin (AMP), amoxicillin (AMO), gentamycin sulfate (GTM), streptomycin sulfate (STM), erythromycin (ERY), tetracycline hydrochloride (TC), and chloramphenicol (CHL).

In general terms, 52% (9 out 17) of the experiments conducted on Gram-positive bacteria resulted in an ABX MIC reduction ≥ 75%, while this behavior was observed for only 26% (8 out of 30) of the tests on Gram-negative bacteria, Table 2. Six of these interactions were found to be synergistic: GTM-EUG-*L. monocytogenes*, GTM-EUG-*S. aureus*, CHL-EUG-*A. baumannii*, STM-EUG-*S. enterica*, TC-EUG-*S. marcescens,* and CHL-EUG-*S. marcescens*. All exhibited fractional inhibitory concentration (ΣFIC) values equal or below 0.50, calculated as described in Section 3.4. On the other hand, only two combinations resulted as antagonistic, which were STM-EUG-*A. baumannii* and CHL-EUG-*K. aerogenes*, both Gram-negative species. Although most of the interactions were additive, it must be highlighted that seven out of these additive combinations, AMP, AMO and TC-EUG-*L. monocytogenes* or GTM and CHL-EUG-*S. agalactiae*, STM-EUG-*S. aureus,* and CHL-EUG-*K. pneumoniae*, resulted in a pronounced decrease in the ABX dose (more than 75%, Table 2).

**Figure 1:**
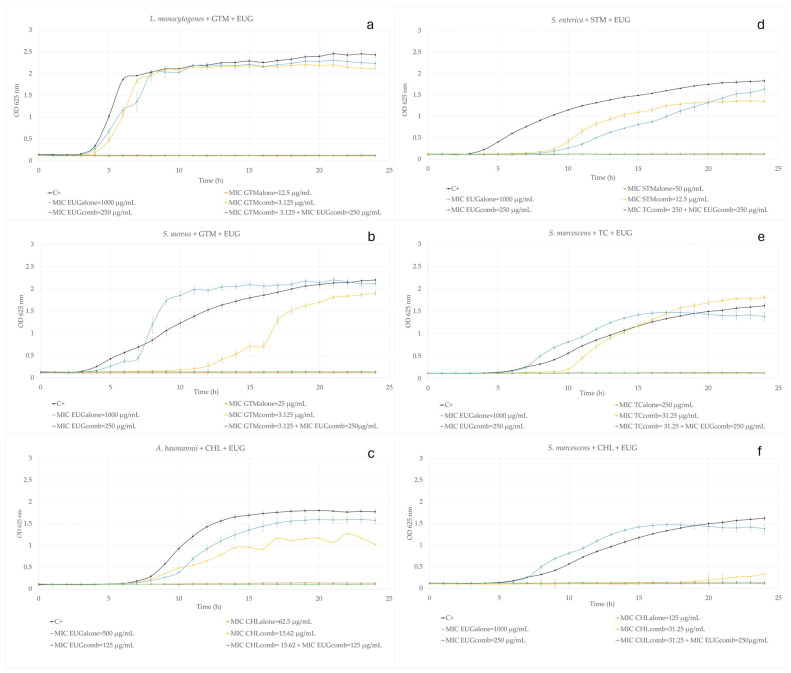
Kinetic study for eugenol (EUG) and (**a**) gentamicin (GTM) on *L. monocytogenes*, (**b**) GTM on *S. aureus*, (**c**) chloramphenicol (CHL) on *A. baumannii*, (**d**) streptomycin (STM) on *S. enterica*, (**e**) tetracycline (TC) on *S. marcescens,* and (**f**) CHL on *S. marcescens* (OD at 625 nm vs. time (h)). C+: curve for positive control. MIC EUG_alone_ and MIC ABX_alone_ are the curves for EUG and ABX, respectively, when each of them was tested alone at their respective MICs. MIC ABX_comb_ is the curve for ABX tested alone but added at its MIC when this and EUG were tested simultaneously. MIC EUG_comb_ is the curve for EUG tested alone but added at its MIC when this and ABX were tested simultaneously. (MIC ABX_comb_ + MIC EUG_comb_) is the curve for the combination of the mixture of ABX and EUG when tested simultaneously at their respective MICs in combination. Data are given as mean ± standard deviation.

Additionally, a comprehensive kinetic growth study was conducted on the six synergistic interactions, as shown in Figure 1, to unravel the behavior of each microorganism over a 24 h period under controlled conditions. It was observed that the combined administration of commercial ABXs and EUG, both at their MIC_comb_ (see Section 3.5 for a definition of MIC_comb_) led to a complete inhibition of bacterial growth across all instances. These graphs also depict the total growth inhibition in experiments when natural and commercial ABXs were individually administered at their respective MIC_alone_ (see Section 3.5 for a definition of MIC_alone_) and the non-inhibition when tested alone but at their respective MIC_comb_. However, even at subinhibitory concentrations of both the ABX and the natural product, some interesting facts can be extracted. The most pronounced effect observed in the graphs is related to the prolongation of the lag-phase. The lag-phase when the ABX was tested alone at its MIC_comb_ was elongated for all experiments except for GTM-EUG-*L. monocytogenes* (Figure 1a) and CHL-EUG-*A. baumannii* (Figure 1c). However, when EUG was tested in these same circumstances, the lag-phase was modified only for *S. enterica* (Figure 1d). This fact was previously observed by Silva et al. [70] on *P. aeruginosa* when EUG was tested at subinhibitory concentrations. They suggested that this behavior could be attributed to interactions between it and primary non-vital target sites of bacteria.

#### 2.2.1. Aminoglycosides

The precise synergistic mechanism between EUG and aminoglycosides remains unclear. However, previous studies suggest that the damage to the bacterial cell envelope induced by EUG may facilitate the entry of aminoglycosides (which use active transport systems to enter bacterial cells [73] and inhibit protein synthesis [74]), thereby inhibiting bacterial growth at lower doses, albeit with variations depending on the microorganism [48,75]. This mechanism would be especially efficient on Gram-positive bacteria, as observed for STM and GTM when acting together with EUG on *S. agalactiae*, *L. monocytogenes,* and *S. aureus*, Table 2, as shown in this work and previous research [75,76]. For them, five of the six tested combinations resulted in an ABX MIC reduction ≥ 75%, while for the Gram-negative bacteria, this trend was observed only in two out of six. Within this group of bacteria, the most significant reduction in the aminoglycoside dosage was seen for STM against *S. enterica*, also displaying synergy (Table 2 and Figure 1d), as previously demonstrated by Liu et al. [49], who noted the antibiofilm action of EUG in combination with STM. Additionally, while not synergistic but additive, EUG demonstrated substantial reductions in the aminoglycoside dosage when combined with STM against *S. agalactiae*, *S. aureus,* and *K. pneumonia*, as well as with GTM against *S. agalactiae* (Table 2). This positions aminoglycosides as the antibiotic class most affected by their combination with EUG. Regarding the antagonistic interaction observed when EUG was combined with STM on *A. baumannii*, EUG might somehow alter the membrane potential [64] and thus lead to a reduction in the active transport of STM into the cell, resulting in the observed antagonistic effect, Table 2.

#### 2.2.2. Beta-Lactams

Aminopenicillins are relatively small and hydrophilic molecules that use porins as essential entry points in these bacteria [77]. Beta-lactams inhibit the activity of penicillin-binding proteins (PBPs), crucial for peptidoglycan cross-linking during cell wall synthesis, weakening the cell wall and altering the osmotic balance [78,79]. This combined with EUG’s membrane-disrupting effects might lead to cell lysis [46,47,48,54,55,56,80,81]. As expected, this effect was stronger in Gram-positive bacteria since the latter has a less complex envelop. Indeed, half of the tested interactions, versus 1 out of 10 for the Gram-negative bacteria, resulted in an ABX MIC reduction ≥ 75%. Although AMO and AMP did not exhibit synergistic interactions with EUG, they approached a value of ΣFIC = 0.5 for *L. monocytogenes*, showing a significant reduction in the ABX dose, although in an additive manner. Previous studies also found that EUG, when combined with AMP and AMO, led to an ABX dose reduction in a synergistic or additive manner on different strains of *E. coli*, *P. aeruginosa*, and *S. Typhimurium* or with AMO on *E. faecalis* [82,83,84]. Our results may be especially significant for bacteria prone to developing resistance to beta-lactams through efflux pumps because the co-administration of ABX and EUG, which is known to inhibit efflux pumps activity, might result in a high intracellular ABX concentration [64,85].

#### 2.2.3. Amphenicols

The mechanism of action of CHL involves the reversible inhibition of bacterial protein synthesis by binding to the 50S subunit of the bacterial ribosome [78,86,87]. It is a broad-spectrum antibiotic with high liposolubility, allowing it to enter bacteria through passive diffusion [87]. The synergistic behavior observed for EUG when combined with CHL on *A. baumannii* (Table 2 and Figure 1c) could involve the inhibition of the AdeABC efflux pump by EUG without increasing the membrane permeability [64]. This could lead to the increased retention of CHL inside the cell, enhancing growth inhibition. Similarly, synergy between EUG and CHL was observed in this study against *S. marcescens* (Table 2 and Figure 1f), proposing a possible shared mode of action involving efflux pump inhibition.

Although there is limited research available on the remaining interactions in the selected microorganisms, EUG was found to decrease CHL MIC also in *E. coli*, *P. aeruginosa,* and *S. enterica* [88], as observed in Table 2. Unfortunately, we have no explanation concerning the antagonistic effect of CHL + EUG on *K. aerogenes*, since nothing is known about the mode of action of EUG in this species.

#### 2.2.4. Tetracyclines

TC is a broad-spectrum antibiotic that inhibits bacterial protein synthesis by binding to the 30S subunit of the bacterial ribosome, specifically at the A site, blocking the attachment of aminoacyl-tRNA molecules [89]. This inhibition prevents the elongation of the polypeptide chain during translation. TC can also interfere with other bacterial processes, including essential metabolic pathways and cell membrane integrity, contributing to its bacteriostatic effect [89]. The mechanism underlying the combined action of TC and EUG remains uncertain, although membrane disruption appears to be a plausible explanation, specifically in *S. marcescens* and *L. monocytogenes*, for which an ABX MIC reduction ≥ 75% was achieved (Table 2 and Figure 1e). While there is limited literature on the combination of TC with EUG against these bacteria, a decrease in the ABX MIC for *E. coli*, *P. aeruginosa*, and *S. Typhimurium* has been previously reported [83,88]. However, we did not investigate these combinations due to their initially low MIC (see Section 3.4. for detailed information).

#### 2.2.5. Macrolides

Macrolides, such as ERY, enter the cells via passive diffusion, inhibit protein chain elongation, and are effective against a broad spectrum of bacteria [87]. When combined with EUG, ERY demonstrated MIC reductions across most tested bacteria, as previously determined on *S. enterica*, *E. coli*, and *S. Typhimurium* [82,83]. Given EUG’s known modifications to the cell envelope, the mechanism of action of the EUG-ERY combination may be similar to that proposed for TC and EUG.

### 2.3. Eugenol Ecotoxicity

The impact of EUG on soil and water environments is not extensively documented. EUG is reported to have effective insecticidal properties and it was approved by the Environmental Protection Agency (EPA) in the USA due to its minimum risk on mammals [90,91]. In this report, some toxicity data are given, mostly on mammals, but there are also data (very few) referring to invertebrates that will be commented on later.

To draw more comprehensive conclusions about EUG ecotoxicity, we have selected different soil and water non-target organisms together with microbiota extracted from water and soil, on which the metabolic effect of EUG was assessed.

#### 2.3.1. Water Ecotoxicity of Eugenol

Water ecotoxicity was analyzed through three indicators: *Vibrio fischeri*, *Daphnia magna,* and a complete water bacterial community, in which a comprehensive analysis of growth and metabolites’ intake was carried out.

- Average Well Color Development (AWCD) of water microbial populations and Community-Level Physiological Profiling (CLPP) after treatment with eugenol.

The taxonomic categorization, spanning from the kingdom to species, was derived through the analysis of 16S rRNA sequences obtained from our river water samples. Across the levels of species, genus, family, order, class, phylum, and kingdom, the proportion of total reads ranged from 94.13% to 99.35%, while for species, it constituted 79.00%, Figure S1 and Table S1. This microbial profile of the water sample is representative of a fluvial environment in the Mediterranean area and similar to that reported by other studies [92,93].

**Figure 2:**
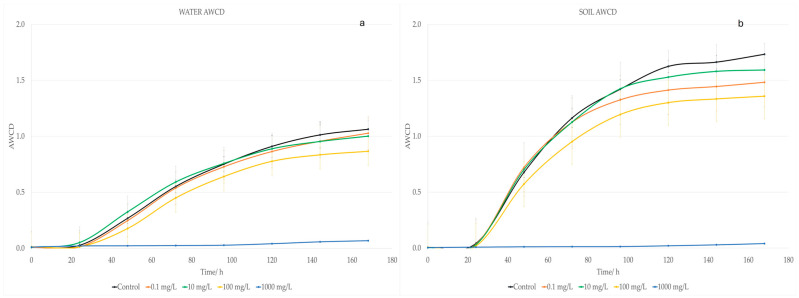
Average well color development (AWCD) of metabolized substrate in Biolog EcoPlates^®^ by (**a**) river water microorganisms and (**b**) soil microorganisms for 168 h exposure to different concentrations of eugenol (shown at the bottom of the figure). Values are compared to a control value as reference (river or soil communities not treated with eugenol, respectively). Each point is an average of three replicates and includes the error bars, representing their standard deviation.

Microbial water communities were exposed to EUG for a 7 day-period on the Biolog Ecoplates^®^ (Figure 2a). According to the statistical analysis, only after 48 h of exposure were significant differences (*p* < 0.01) found between the control and an EUG concentration of 1000 mg/L. For the lowest concentrations (0.1, 10, and 100 mg/L), no significant decrease in the ability to degrade carbon sources was observed, since all values were similar to those of the control group.

**Figure 3:**
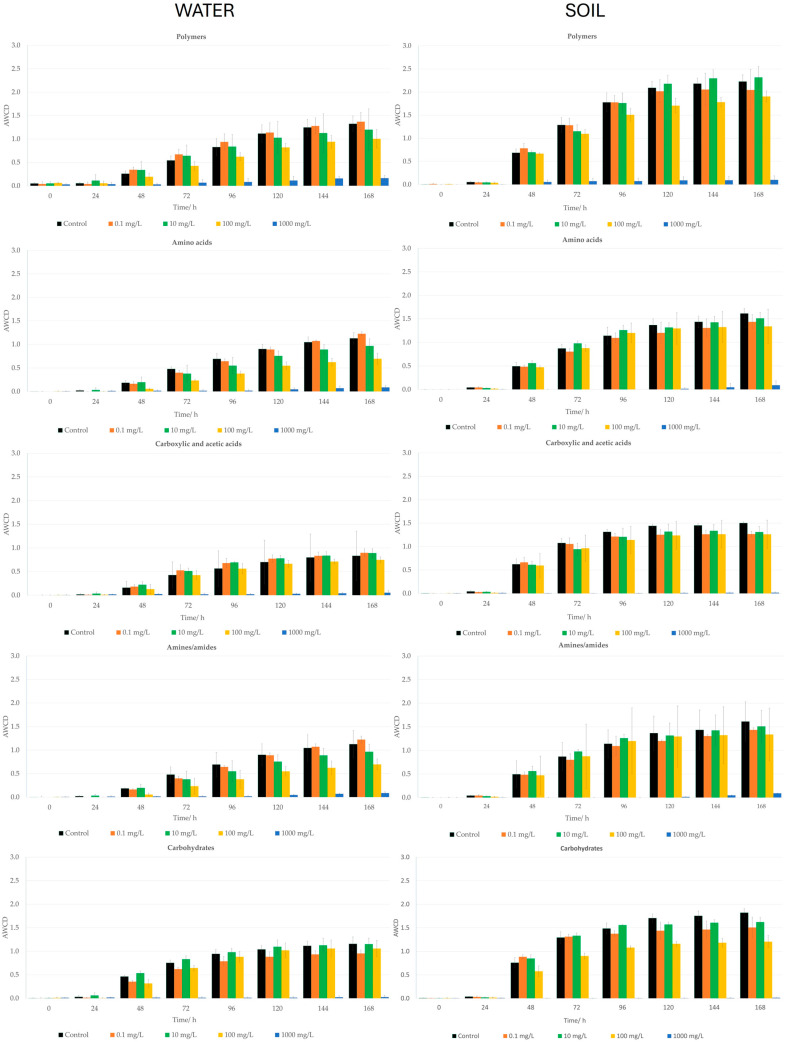
Metabolic profile of water and soil samples for 168 h exposure to different eugenol concentrations and control. Bars represent the AWCD growth for each group of metabolites of bacteria. Each value is an average of three replicates and includes the error bars representing their standard deviation.

The microbial CLPP is understood as the metabolic capacity for degrading different carbon sources after toxic exposure [94]. This method assesses the whole impact of toxic substances on these communities’ metabolism overall or by the group of metabolites. The changes in the CLPP allow us to identify the impact of EUG on the capacity of water microorganisms to metabolize the 31 most frequent organic carbon sources [94]. For a better understanding of the metabolic changes, these carbon sources were grouped into polymers, amino acids, amines/amides, carbohydrates, and carboxylic and acetic acids [94]. These results are shown in Figure 3 and Table S2.

The metabolic ability of the water microbial communities is barely affected by EUG with respect to the control. As observed when analyzing the AWCD, only the highest concentration of EUG substantially changed the metabolic profile of these microorganisms. In fact, statistical differences (*p* < 0.01) were detected between the control and 1000 mg/L for four of the five groups of metabolites (carbohydrates, polymers, amino acids, and amines/amides) from 48, 96, 120, and also 120 h, respectively. For the amino acids group, there was also significant differences between the control and 100 mg/L of EUG from 96 h. For the acids, no statistical difference was detected at any time for any of the EUG doses tested.

When comparing the effects of EUG and commercial ABXs (the ones used in the antimicrobial experiments) [93] on water microbiota, our EUG data revealed a weaker impact on AWCD. EUG and commercial ABXs caused a significant reduction in AWCD only at 1000 mg/L, but GTM, CHL, and TC also induced significant decreases at 100 mg/L [93]. Regarding CLPP, EUG showed substantial metabolic differences compared to the control at 1000 mg/L for all metabolic groups except amino acids, which exhibited differences at 100 mg/L. In contrast, commercial ABXs had more pronounced effects on the microbial metabolism at all tested concentrations (from 0.1 to 1000 mg/L), with few exceptions such as AMP and CHL at lower concentrations [93]. Our findings suggest that bacterial communities with high taxonomic diversity, like those in our samples, are less affected by EUG exposure compared to commercial ABXs [93,95]. This could be attributed to antibiotic-degrading resistance mechanisms present in some community members, such as *Pseudomonas* sp. [96] which is present in our sample (Table S1), which may degrade EUG more efficiently than commercial ABXs. Consequently, while some species may be impacted, they may be replaced by resistant ones, resulting in no overall impact on population growth, as measured by AWCD, except at very high concentrations.

- Effect of EUG on water non-target organisms: *Vibrio fischeri* and *Daphnia magna.*

**Figure 4:**
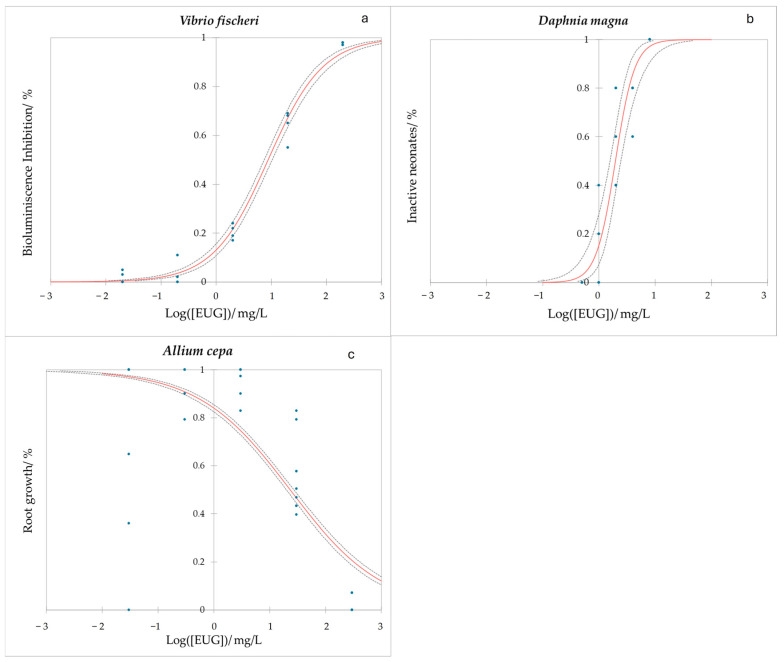
Dose–response curve for the (**a**) bioluminescence assay for *Vibrio fischeri* after 30 min exposure to eugenol (curves are the average of 4 replicates); (**b**) *Daphnia magna* test after 24 h exposure to eugenol (curves are the average of 5 replicates); and (**c**) *Allium cepa* test after 72 h exposure to eugenol (curves are the average of 12 replicates). Red line is the model, blue dots are experimental data, and dashed lines are the inferior and superior confidence limits (95%).

A bioluminescence inhibition assay on *V. fischeri* and its dose–response curve revealed toxicity for these Gram-negative bacteria, as can be appreciated in Figure 4a. The experiment’s significance was high (*p* < 0.0001) and both EC_10_ and EC_50_ values were measured for the 30 min exposure test, being 0.708 mg/L (CI: 0.533–0.906) and 8.778 mg/L (CI: 7.454–10.370), respectively.

The *D. magna* immobilization test dose–response curve can be observed in Figure 4b. The results of the 24 h exposure test, with a high level of significance (*p* < 0.0001), revealed EC_10_ and EC_50_ values of 0.824 mg/L (CI: 0.496–1.098) and 1.963 (CI: 1.571–2.444) mg/L, respectively.

*V. fischeri* and *D. magna* are both valuable indicators of ecotoxicity, with the former’s bioluminescence enhancing its sensitivity to environmental stressors [97]. Lal et al. [98] and Gueretz et al. [99] found lower EC_50_ values for EUG in these organisms compared to our study, but their analysis lacked statistical rigor and employed fewer concentrations. In our study, with five doses and statistical analysis, slightly higher EC_50_ values were found for both organisms, aligning closely with the findings of Baker and Grant (2018) for *V. fischeri*.

Regardless of the reference values chosen, EUG demonstrates significantly lower EC_50_ values compared to the ABXs examined in our study. For instance, EC_50_ values for AMO ranged from 150 mg/L [100] to 4000 mg/L [101], for AMP from 163 mg/L [100] to 1056 mg/L [102], for TC from 6.70 mg/L [103] to 173.8 mg/L [104], and for CHL from 20.68 mg/L to 1086 mg/L [105,106,107,108,109] in immobilization tests lasting 24–48 h. The highest EC_50_ value recorded was for GTM (>10,000 mg/L) [110], while the lowest was 8.21 mg/L for STM [100]. No acute ecotoxicity was observed for ERY on *V. fischeri*. In the case of *D. magna*, EC_50_ values for AMO ranged from >1000 mg/L [102] to 6950 mg/L [111], for AMP were also >1000 mg/L, and for GTM and STM ranged from 875.5 mg/L [110] to 947 mg/L [112]. Reported EC_50_ values for ERY [113] and CHL [114,115] were lower than those for AMO and AMP, but higher than those for EUG. Only Havelkova et al. [103] provided a TC EC_50_ value of 8.16 mg/L in a test with a longer exposure duration compared to ours. EUG continues to exhibit higher acute toxicity on non-target organisms such as *D. magna* compared to commercial ABXs. *D. magna* exhibits greater sensitivity to EUG compared to *V. fischeri*, consistent with prior research [99]. Microcrustaceans generally appear more vulnerable to pollutants than bacteria [99]. While the mode of action of EUG on *V. fischeri* remains unclear, it may involve mechanisms similar to those affecting other Gram-negative bacteria. Studies suggest that EUG acts as an ion channel blocker in *D. magna*, disrupting the proper function of its myogenic heart and causing a dose-dependent increase in the duration of muscle relaxation [116,117,118,119]. Further research is necessary to fully understand EUG’s mode of action.

#### 2.3.2. Soil Ecotoxicity of Eugenol

Soil ecotoxicity was analyzed through a complete soil bacterial community, in which a comprehensive analysis of growth and metabolites intake was performed. In addition, soil ecotoxicity was also assessed on two indicators: *Eisenia foetida* and *Allium cepa*.

- Average Well Color Development (AWCD) of soil microbial populations and Community-Level Physiological Profiling (CLPP) after treatment with eugenol.

Within the hierarchy of family, order, class, phylum, and kingdom, the proportion of total reads ranged from 93.71% to 99.49%, while for genus and species, it was 90.31% and 63.09%, respectively (Figure S2 and Table S3). The microbial profile detected in our soil sample is coherent with the type of microorganisms expected in a sample of these characteristics, as previously described by other authors [92,120,121].

A similar effect as the one found in water was observed for the soil sample (Figure 2b) when AWCD was plotted versus a period of 168 h. From 24 to 168 h, statistical differences between the control and 1000 mg/L were found (*p* < 0.01). However, at 168 h, the AWCD values between the control group and the dose of 100 mg/L were also significantly different.

Figure 3 provides the data of the metabolic changes of the soil microorganisms after exposure to EUG for 168 h. For polymers, acids, and amino acids, significant differences (*p* < 0.01) between the control and EUG at 1000 mg/L were found from 24 h. However, significant differences for amines/amides and carbohydrates were observed from 48 h and for this last group, significant differences between the control and an EUG dose of 100 mg/L was also detected after 72 h.

To our knowledge, previous studies have not investigated the impact of EUG on soil microorganism communities. However, Pino-Otín et al. [122] documented the AWCD and CLPP over time of different concentrations of AMO, AMP, STM, GTM, TC, ERY, and CHL on the soil microbiota. All commercial ABXs led to decreased AWCD at both 100 and 1000 mg/L, except for STM, which only reduced it at 100 mg/L [122]. In contrast, EUG affected the soil microbiota mainly at 1000 mg/L, indicating lower ecotoxicity compared to commercial ABXs. Pino-Otín et al. [122] observed the decreased metabolism of all five carbon sources with commercial ABXs, occurring either after 48 or 72 h across all concentrations. However, the influence on different carbon source groups varied, with amines and polymers showing the most significant changes. Conversely, EUG reduced the metabolism across all five groups equally, but only at 1000 mg/L. Similar to the water samples, soil bacterial communities exhibited higher resistance to EUG when composed of different species compared to single-species communities [95].

- Soil non-target organisms: *Eisenia foetida* and *Allium cepa*.

A lethal dose of EUG for *E. foetida* was assessed by the 14-day mortality test, with this compound being toxic for its survival at very high concentrations. Up to 100 mg/L, the product does not induce mortality effects on *E. foetida*.

After the 72 h exposure of *A. cepa* roots to EUG, its elongation was shortened, meaning that the EUG resulted as being toxic for the bulbs. The experiment showed a high degree of significance (*p* < 0.0001) and the results are shown in Figure 4c. The EC_10_ values were measured as 1484.190 (CI: 1112.860–2039.888) mg/L and the EC_50_ values correspond to 23.116 (CI: 20.061–26.743) mg/L.

*E. foetida*, a common earthworm species, plays a crucial role in soil quality through its digging, feeding, and nutrient cycling activities, contributing to the soil structure and aeration. Its presence and behavior serve as indicators of soil health and contamination due to its susceptibility to pollutants, such as heavy metals [123,124,125,126]. However, limited information exists on the effects of natural compounds on this species. In our study, the LC_50_ for EUG was > 100 mg/kg, consistent with the findings by Almadiy and Nenaah [127], who observed no toxicity within a similar concentration range. Few studies have investigated the toxicity of antibiotics on *E. foetida*; notably, TC effects were studied over a longer duration (56 days), yielding an LC_50_ value of 2735 mg/kg [103]. *E. foetida* exhibits notable resistance compared to other indicators, likely due to its complex multicellular nature.

*A. cepa* is recognized as an indicator of soil, air, and water contamination, owing to its sensitivity to pH levels and nutrient requirements [128,129]. The development and yield of onions depend directly on soil quality, particularly the presence of key metabolites like nitrogen, sulfur, and potassium. The responses of onions to these factors can indicate the soil nutrient status and deficiencies [130]. Despite numerous studies on *A. cepa*, the effects of EUG on its roots remain unexplored, except for one study by Gogoi et al. [131], who found no toxicity of the essential oil containing EUG. Our study similarly observed the low toxicity of EUG on *A. cepa* root elongation (EC_50_ = 23.116 mg/L), possibly due to its poor water solubility. In contrast, experiments with clove oil solutions containing EUG showed strong phytotoxicity on weeds, but they used an adjuvant (nonionic surfactants and paraffinic oil blend) to help dissolve it [132]. While no specific ecotoxicity tests for ABXs on onions have been conducted to our knowledge, studies have examined how STM and TC affect these plants at the chromosome level [133,134,135].

Our ecotoxicity findings indicate that EUG poses less harm to soil and water microbiota compared to commercial ABXs, possibly due to the faster metabolism by certain bacteria present in the samples. EUG demonstrated relatively low acute ecotoxicity for *A. cepa* and *E. foetida*; however, direct comparisons with commercial ABXs are unavailable. In contrast, EUG exhibited higher acute ecotoxicity than some commercial ABXs for *V. fischeri* and *D. magna*. Nonetheless, the environmental impact of EUG would be minimal compared to commercial ABXs in case of being used as an adjuvant, as the proportion excreted into the environment without a mammalian metabolism is less than 0.1%, whereas for various ABXs, it ranges from 5% to 100% in human urine [136,137,138,139,140,141,142,143].

## 3. Materials and Methods

### 3.1. Reagents

Commercial antibiotics used for this research were AMP (CAS 69-53-4, purity > 96%), AMO (26787-78-0, purity > 96%), GTM (1405-41-0, purity > 96%), STP (3810-74-0, purity > 96%), ERY (114-07-8, purity 97.5%), TC (64-75-5, purity 99.2%), and CHL (56-75-7, purity 99.6%). AMP and AMO were acquired from Sigma Aldrich (Burlington, VT, USA), while GTM, ATP, ERY, and CHL from Acofarma, (Barcelona, Spain). On the other hand, the natural compound EUG (CAS 97-53-0, ≥98.5%) was also purchased from Sigma Aldrich (Burlington, VT, USA) and DMSO (CAS 67-68-5, >99.7%) from Fischer Bioreagents (Pittsburgh, PA, USA).

### 3.2. Bacterial Strains Growth

Information concerning the strains used in this research and their growth conditions is shown in Table 3. Each strain was acquired lyophilized from Thermo Scientific (Waltham, MA, USA) in culture loops. The original strains were frozen in Cryoinstant Mix cryotubes (Deltalab, Barcelona, Spain) in accordance with the manufacturer’s recommendations to prevent mutations. After that, they were kept at −80 °C (Froilabo, Trust −80 °C, Collégien, France) and rehydrated in accordance with each microorganism’s technical information sheet for use [144], for which a summary is offered in Table 3.

As previously described by Ferrando et al. [2], the bacterial inoculum was re-cultured from the cryotubes and incubated (J. P. Selecta, Barcelona, Spain) for 24 h in the conditions necessary for each optimum bacterial growth (Table 3) before any microorganism was used. After that, the culture was left overnight to reach the necessary bacterial optical density of 2.5 × 10^8^ CFU/mL or 0.5 McFarland scale [145].

### 3.3. Minimum Inhibitory Concentration (MIC)

The MIC of EUG was determined on each bacterial strain according to the Clinical and Laboratory Standards Institute [145,146,147]. A stock solution of EUG was prepared by dissolving it into sterile water with 5% of DMSO, giving a concentration of 4000 μg/mL. The MIC of the ABXs cited in Section 2.1. and the toxicity of DMSO on the strains used in this work were analyzed in a previous study by Ferrando et al. [2]. In that study, bacterial growth experiments were conducted in aqueous DMSO solutions with concentrations ranging from 0.04% to 20% (*v/v*). The objective was to identify the DMSO concentration that would allow the proper dissolution of the natural compound (cinnamaldehyde in that work, EUG in the current research) without adversely affecting bacterial growth. This optimal concentration was found to be 2.5%. At this concentration, bacterial growth was not affected for all bacteria except for *P. mirabilis*, which exhibited greater sensitivity to DMSO. A DMSO concentration of 0.16% would have been required to avoid the growth inhibition of *P. mirabilis*, but at this concentration, (as with cinnamaldehyde) it was not soluble. As mentioned in Ferrando et al. (2024) [2], not all possible ABX–EUG–Bacteria combinations were tested. Only ABXs with an MIC for a given bacteria type above 10 μg/mL were included in the study, whereas bacteria sensitive to DMSO concentrations below 2.5% were excluded because of the impossibility to solubilize EUG. According to these criteria, 47 combinations of ABX–EUG–bacteria were selected for the checkboard assay.

To summarize, 100 μL of broth and 100 μL of EUG stock solution were added to each well of a 96-well round-bottom plate. After the bacterial suspension was adjusted using the 0.5 McFarland scale, 10 μL of it was added to the samples in two-fold dilutions. Additionally, negative and positive controls were introduced. The standard growth of bacteria in the absence of an antimicrobial agent was considered the positive control. The sole purpose of the negative control was to make sure that the culture broth was free of contamination or microbial development. Using a BioTekTM Synergy H1 Hybrid multimode microplate (Agilent, Madrid, Spain), absorbance was measured at 625 nm, at the optimal temperature for each bacterium, following the 24 h incubation period. Each experiment was carried out three times.

### 3.4. Bacterial Checkboard Assay

The nature of the interactions between the natural chemical and each commercial antibiotic were investigated following the checkerboard assay technique [2,148,149,150]. For this analysis, commercial ABX stock solutions were prepared by diluting them in sterile water at a concentration of 4 times their respective MICs. The procedure was the same for EUG, but the sterile water contained 5% of DMSO to guarantee its dissolution, as described in the previous section.

Briefly, from the first to the seventh columns of the 96-well plate (round-bottom), EUG serial two-fold dilutions were added, while ABX solutions were added from rows A to G. As a result, the concentration of both types of antimicrobial drugs tested varied by well. A1 was the most concentrated well, while G7 was the least. After applying repeated two-fold dilutions to the plate, 10 μL of bacterium inoculum (0.5 McFarland) was added. Positive (antimicrobial-free bacterium) and negative (culture media without bacteria) controls were also constructed.

To examine the interactions, two types of Fractional Inhibitory Concentration indices (FIC) were obtained, FIC A and FIC B (Equations (1) and (2), respectively).

FIC A = (MIC of A in combination with B)/(MIC of A alone)(1)

FIC B = (MIC of B in combination with A)/(MIC of B alone)(2)

where A and B were each commercial ABX and EUG.

The ∑FIC was calculated as the addition of both FICs. This value let us classify the interactions between the commercial ABX and EUG as follows: synergistic if ∑FIC ≤ 0.5, additive if ∑FIC was between 0.5 and 4, and antagonistic if ∑FIC ≥ 4 [29,151,152].

Experiments were carried out in triplicate and in sterile flow chambers (Model MSC Advantage 1.2).

### 3.5. Bacterial Kinetic Growth Assay

The procedure used was somewhat modified from that given by the Clinical and Laboratory Standards Institute [153,154,155]. Wells in a 96-well plate (round-bottom) were filled with: (i) a commercial ABX solution at its MIC when tested alone (MIC_alone_), (ii) a commercial ABX solution at its MIC when tested in combination with the natural product (MIC_comb_), (iii) a natural compound solution at its MIC when tested alone, (iv) a natural product solution at its MIC when tested in combination with the commercial ABX, and (v) a solution made of natural compound and commercial ABX both at their respective MICs when tested in combination [2,45,156].

At each bacterial optimum temperature (Table 3), absorbance was measured every hour for 24 h at 625 nm using a SPECTROstar Nano from BMG Labtech (Madrid, Spain). Experiments were carried out in triplicate, and all results were presented as mean ± standard deviation.

### 3.6. Average Well Color Development (AWCD) Tests of Soil and Water Microorganisms

#### 3.6.1. Water Samples

Water samples were collected from the Gállego River (41°41′57″ N, 0°49′1″ W, Zaragoza, Spain) in June 2022. Procedures were carried out in situ according to standard procedures [157]. The water temperature was 17 °C and its pH was neutral. Its physico-chemical characteristics are given in Table S4.

Five liters of collected river water were used to isolate microorganisms for the genetic analysis using a 0.22 μm cellulose nitrate filter (Sartorius, Aubagne, France). The water was then resuspended in a sterile Falcon tube with 50 mL of sterilized phosphate-buffered saline (PBS) and centrifuged for 10 min at 5000× *g* to extract microorganisms for genetic analysis. The supernatant was removed, and the pellet was stored at −80 °C prior to sequencing.

A 70 μm nylon filter (Becton Dickinson, Madrid, Spain) was used to filter 1 L of river water to remove debris, and then stored at 4 °C until it used for further experiments.

#### 3.6.2. Soil Samples

The procedure used for soil preparation was carried out according to earlier publication [158]. Sample was obtained in June 2022 from a contaminant-free crop field (CITA, Zaragoza, Spain). The sample was first sieved to eliminate any contaminants larger than 2 mm, and it was then kept in darkness in sterile plastic bags. In a laminar flow biological safety hood to guarantee sterility (Model MSC Advantage 1.2, Thermo Fischer Scientific, Waltham, MA, USA), 95 mL of distilled, filtered (Sterifix^®^ 0.2 μm, Fischer Scientific, Waltham, MA, USA) water were added to 10 g of soil to begin soil microbial extraction, while mild magnetic stirring was performed for 30 min. Six falcon tubes were filled with 10 mL of the mixture’s supernatant after it had been allowed to stand for 1 h. The mixture was then sonicated for 1 min and centrifuged (at 1000× *g* for 10 min at 7 °C) with a Thermo Scientific Heraeus biofuge Primo R centrifuge (Waltham, MA, USA). The supernatants were collected and combined in a sterile tube. The pellets in the falcon tubes were then filled with 10 additional mL of filtered water and centrifuged again. This operation was repeated five times. The 60 mL finished leachate was then filtered to eliminate contaminants using a 70 μm nylon filter (Becton Dickinson, Madrid, Spain).

#### 3.6.3. Biolog Ecoplates Preparation

Using Biolog EcoPlates^TM^ (Newark, NJ, USA) which contained three replicates of the 31 most important organic substrates for microbe metabolism, along with a water control, the microbial level physiological profile—defined as the metabolic capacity for degrading various carbon sources after substance exposure—was determined [159]. This approach evaluates the full effect that potentially harmful compounds may have on the metabolism of these communities [122,160,161]. At each concentration of EUG (0.1, 10, 100, and 1000 mg/L), wells of the Biolog EcoPlates^TM^ were filled with 75 mL of soil leachate or processed water and 75 μL of EUG solutions. Three duplicates of each concentration were tested. The dilutions’ ultimate pH ranged from 6 to 7. J.P. Selecta (Barcelona, Spain) plates were sterilely incubated for 7 days at 25 °C. With the use of Gen5TM data analysis software (version number 1.08.4) and a BioTek Synergy H1 microplate reader (Agilent, Madrid, Spain), each well’s optical density (OD) was measured at t = 0 and every 24 h at 590 nm.

With the OD values, the Average Well Color Development (AWCD) was calculated as follows [162,163]:

AWCD = ∑(AbsW − AbsC)/31(3)

where AbsW is the absorbance (or optical density) of each well with the carbon source and AbsC is the absorbance of the control well without it.

According to comparable patterns of use, the 31 substrates were grouped into five functional classes: amines/amides, amino acids, carboxylic and acetic acids, polymers, and carbohydrates [94]. Once this was completed, Equation (3) was applied to calculate the AWCD of each metabolic group [163].

Repeated measures ANOVA test was performed to analyze the evolution of absorbance over time, with post hoc comparisons by Sidak if statistical significance was reached. ANOVA was carried out separately for each concentration and in a comparative way with the control as a factor. SPSS 28.0 software was used with a threshold value of *p* = 0.01 to accept or reject null hypothesis.

### 3.7. Genetic Analysis of Water and Soil Sample

The prefiltered solution underwent subsequent filtration employing Sartorius (Madrid, Spain) 0.2 µm cellulose nitrate filters, which were thoroughly rinsed with PBS solution adjusted to a pH of 7.5. This resultant solution was then collected into Falcon tubes and subjected to centrifugation at 5000× *g* for a duration of 10 min. Subsequently, supernatants were meticulously decanted, and pellets were preserved by freezing at −80 °C for subsequent genetic analysis utilizing Froilabo (Paris, France), Trust −80 °C equipment [122].

A quantity of 50 ng of DNA underwent amplification according to the 16S Metagenomic Sequencing Library Illumina 15,044,223 B protocol (Illumina, Paris, France) within the facilities of ADM BIOPOLIS laboratories (Parc Científica, Universitat de Valencia). In summary, the initial amplification phase involved the utilization of primers that incorporated: (1) a universal linker sequence facilitating the incorporation of amplicons with indexing and sequencing primers through the Nextera XT Index kit (Illumina, Paris, France), and (2) universal primers targeting the 16S rRNA gene [164]. In the final amplification step, indexing sequences were integrated. The quantification of the 16S-based libraries was conducted via fluorimetry employing the Quant-iT™ PicoGreen™ dsDNA Assay Kit (Thermofisher, Waltham, MA, USA).

The pooling of libraries occurred prior to sequencing on the MiSeq platform (Illumina, Paris, France) utilizing a configuration of 300 cycles for paired reads. The determination of pool size and quantity was conducted utilizing the Bioanalyzer 2100 (Agilent, Madrid, Spain) and the Library Quantification Kit for Illumina (Kapa Biosciences, Solo, Norway), respectively. A PhiX Control library (v3) (Illumina, Paris, France) was blended with the amplicon library at an anticipated ratio of 20%. Subsequently, sequencing data became available within an approximate timeframe of 56 h. Image analysis, base calling, and data quality assessment were executed on the MiSeq instrument utilizing MiSeq Control Software (MCS v3.1).

### 3.8. Vibrio Fischeri Bioluminiscence Assay

Purchased lyophilizate bacteria from Macharey-Nagel (ref. 945 006) (Fischer Scientific, Waltham, MA, USA) were kept frozen at −18 °C. The protocol followed was UNE-EN ISO: 11348-3:2007 [165]. Then, lyophilized *V. fischeri* bacteria were rehydrated with the reactivation solution provided by the company and stored at 4 °C for 5 min.

Diluting pure EUG in an aqueous solution of 20 g/L NaCl yielded a stock solution of 4000 mg/L. Using the same solvent, successive dilutions of this were created, that is, 0.4, 4, 40, and 400 mg/L. The sample was strongly stirred for proper oxygenation and its pH was measured to make sure it stayed within the prescribed range (6–8.5). In addition, the appropriate volume of culture media (about 10 mL, purchased from Macharey-Nagel, reference 945 006), was added to the freeze-dried vial to create the bacterial solution. Four replicate measurements were taken for each sample dilution, and all samples and solutions were maintained at a temperature of 15 °C ± 1 °C.

The goal of the experiment was to measure the bacterial solution’s bioluminescence following a brief (10 min) period of rest. Then, aliquots of EUG serial dilutions were added to a volume of the bacterial solution equal to 1 mL and the change in bioluminescence was assessed after 30 min of exposure. As a result, the concentrations tested (0.2, 2, 20, 200, and 2000 mg/L) were half of the serial dilutions described above.

Using the XLSTAT (2014.5.03) program, the EC_50_ and EC_10_ values—effective concentrations of EUG that suppressed bioluminescence by 50% and 10%, respectively—and associated Confidence Intervals (CI) were calculated with the XLSTAT (2014.5.03) software from the dose–response curves for *V. fischeri*.

### 3.9. Daphnia Magna Assay

Tests for *D. magna* (ref. DM121219, water flea, from Vidra Foc, Zaragoza, Spain) were conducted in accordance with standardized procedures [166,167] and the Daphtoxkit FTM magna (1996) operating recommendations. The planktonic crustaceans were kept briefly, until usage, at 5 °C. *D. magna* eggs were incubated for 72 h between 20 and 22 °C in a TOXKIT model CH-0120D-AC/DC incubator (provided by ECOTEST, Valencia, Spain) with 6000 lx illumination. Spirulin provided in the Daphtokit was added to the crustaceans two hours before being exposed to EUG.

EUG was evaluated at final concentrations of 0.1, 1, 10, and 100 mg/L in sterile freshwater [167]. Additionally, this water served as a negative control. A 0.1 M NaOH solution was used to raise the pH to a range of 7–7.5. Each concentration was evaluated with five replicates of five organisms each. Daphnids were cultured at the required concentrations for 24 h in complete darkness at 20–22 °C. When gently agitated for 15 s, organisms were deemed immobile if they were unable to swim.

Effective concentration values of EUG resulting in 50% and 10% (EC_50_ and EC_10_, respectively) immobilization (inactive neonates) and their Confidence Intervals (CI) were obtained from the dose–response curves for *D. magna* mobility tests using the XLSTAT (2014.5.03) software, as mentioned in Section 3.7.

### 3.10. Eisenia Foetida Assay

Adult *E. foetida* earthworms were purchased from Todo Verde (Ourense, Spain) and kept in sphagnum peat substrate (Spanish Flowers Company, Zaragoza, Spain) following recommendations from the provider for their optimum development. A controlled temperature of 18 to 25 °C, pH = 7.5 to 8, and 80% to 85% humidity for 15 days prior to testing was ensured. The experiments were conducted using standardized procedures [168]. Earthworms that were at least 2 months old, had clitella, and weighed between 300 and 600 mg each were chosen for the tests [158,169].

Tests were conducted in 750 cm^3^ plastic jars with lids to avoid animal scape and to ensure the proper humidity. To allow ventilation and oxygen delivery, the jar lids were pierced with holes. The jars were filled with 750 g (wet weight) of standardized [168] soil substrate, which was made up of industrial fine sand, sphagnum peat, and kaolin clay in a 7:1:2 ratio, respectively. Kaolin clay and sand were acquired from Imerys Ceramics (Civita Castellana, Italy), while sphagnum peat was purchased from Verdecora Vivarium (Zaragoza, Spain). Weighing the sample and drying it to a constant mass at 105 °C for 24 h allowed us to calculate the mixture’s water content. Deionized water was added to the medium and carefully mixed to adjust the total moisture content to 35–45% of the dry weight of the soil.

Ten earthworms and EUG at different concentrations (0.1, 1, 10, 100, and 1000 mg/kg) were added to the jars and left for 14 days in a regulated environment (20 ± 2 °C, 80–85% relative humidity, and 400–800 lx of light). Each concentration was tested three times and negative controls without EUG were also prepared.

Lethal concentration values of EUG LC_50_ and LC_10_ and their Confidence Intervals (CI) were obtained, as in Section 3.7, from the dose–response curves for *E. foetida* tests using the XLSTAT (2014.5.03) software.

### 3.11. Allium Cepa Assay

Bulbs of *A. cepa* (variety Stuttgarter Riesen de 14/21) were acquired from Fitoagrícola Company (Castellón de la Plana, Spain) and stored until use in a dry environment, between 10 and 20 °C in the dark. The young bulbs were peeled before the test, preventing damage to the root ring.

*A. cepa* acute toxicity experiments were carried out according to specific standardized procedures [170], which measured root elongation after 72 h of exposure to the test chemical [158]. Mineral water from Aguas de San Martín de Veri S.A. (San Martín de Veri, Spain) was purchased and used as the growth medium for the bulbs. This medium was placed in 15 mL tubes, as it contains an adequate amount of Ca^2+^ and Mg^2+^ (https://www.veri.es/es/el-producto accessed on 12 May 2024). In ecotoxicological experiments, twelve duplicates of each EUG concentration (0.2, 2, 20, 100, and 500 mg/L) were employed. Only water was used for the negative controls. The bulbs were placed in the top of each 15 mL tube and grown for 72 h at 25 °C in a dimly lit space. The tested solutions were renewed every 24 h.

As in Section 3.7, using the XLSTAT (2014.5.03) program, the EC_50_ and EC_10_ values—effective concentrations of EUG that cause 50% and 10%, respectively, of root growth—as well as their Confidence Intervals (CI) were calculated from the dose–response curves for *A. cepa*.

## 4. Conclusions

EUG exhibits antimicrobial activity against various Gram-positive and Gram-negative bacteria, with observed MIC values ranging from 500 to 2000 μg/mL using the microdilution method. Our study is the first to report MIC data for any strain of *B. subtilis*, *K. aerogenes,* and *P. aerogenes* and it is also the first one to provide MIC values obtained through the microdilution method on specific strains never tested before of *L. monocytogenes*, *S. agalactiae*, *S. aureus*, *A. baumannii*, *E. faecalis,* and *K. pneumoniae*. No correlation between the bacterial type and the MIC value was observed. Combination studies with commercial ABXs (ERY, STM, GTM, CHL, TC, AMO, and AMP) using the checkboard assay and the kinetic bacterial growth inhibition study revealed significant findings. Six of the interactions resulted in synergy, particularly with GTM, CHL, STM, and TC, across both bacterial types, with MIC reductions ranging from 75% to 88%. Additive interactions predominated (39 out of 47), allowing for significant reductions in ABX consumption (50% to 98%), suggesting their importance for future research. Only two interactions were found to be antagonistic.

Although an antimicrobial adjuvant is not expected to exhibit such activity, the moderate–low bacterial inhibition capacity of EUG should not be considered an impediment to its potential use as such an adjuvant. EUG–ABXs combination results show its remarkable ability to reduce the ABXs’ dose while maintaining their inhibitory efficacy on pathogenic bacteria.

In terms of acute ecotoxicity, EUG demonstrated a lower impact on soil non-target organisms, namely *E. foetida* and *A. cepa*, compared to commercial ABXs. Additionally, its effect on water and soil microbiota, which are more indicative of potential ecosystem effects, was weaker than that of commercial ABXs. Notably, significant effects of EUG were observed only at 1000 mg/L, whereas commercial ABXs showed effects at lower concentrations. However, only aquatic organisms such as *D. magna* and *V. fischeri* displayed more harmful effects from EUG compared to commercial ABXs. Nevertheless, as less than 0.1% of EUG is excreted by humans without undergoing metabolization (in contrast to an average of 50% for commercial ABXs (as mentioned in Section 2.3.2—Soil non-target organisms), the use of EUG as being antimicrobial and/or an adjuvant poses minimal ecotoxicological risk when compared to commercial ABXs.

In conclusion, our study demonstrates that EUG combined with commercial ABXs exhibits promising antimicrobial activity against clinically relevant Gram-positive and -negative bacteria, suggesting its potential as an antibiotic adjuvant. Moreover, our findings indicate that EUG presents minimal ecotoxicological risk to both terrestrial and aquatic environments, offering valuable insights for its potential use in antimicrobial therapies while considering environmental sustainability.

## Figures and Tables

**Table 1:** Minimum inhibitory concentration (MIC_alone_ (μg/mL)) of eugenol when tested alone on the selected bacteria. Experimental data (Exp) and data found in the literature (Lit).

MIC_alone_ (μg/mL)			
Gram	Bacteria	EUG (Exp)	EUG (Lit)
+	*Bacillus subtilis* (ATCC 6633)	2000	-
	*Enterococcus faecalis* (ATCC 19433)	2000	-
	*Listeria monocytogenes* (ATCC 7644)	1000	-
	*Streptococcus agalactiae* (ATCC 12386)	1000	-
	*Staphylococcus aureus* (ATCC 9144)	1000	-
−	*Acinetobacter baumannii* (ATCC 19606)	500	-
	*Escherichia coli* (ATCC 25922)	1000	>2000 *^a^* 197.4 *^b^*
	*Klebsiella aerogenes* (ATCC 13048)	500	-
	*Klebsiella pneumoniae* (C6)	500	-
	*Pasteurella aerogenes* (ATCC 27883)	500	-
	*Proteus mirabilis* (ATCC 35659)	-	-
	*Pseudomonas aeruginosa* (ATCC 27853)	2000	>2000 *^a^* >1000 *^c^*
	*Salmonella enterica* (ATCC 13311)	1000	525 *^d^*
	*Serratia marcescens* (ATCC 13880)	1000	460 *^e^*

Experimental data (Exp.) were obtained applying the microdilution method. Data extracted from the literature (Lit.) for the same strains are offered for both micro- and/or macrodilution methods. (-): Data not found in Lit. *^a^* Ref [40]; *^b^* Ref [41]; *^c^* Ref [42]; *^d^* Ref [43]; *^e^* Ref [44].

**Table 2:** MICs for commercial antibiotics and eugenol when tested in combination (MIC_comb_ (μg/mL)), Ratio [EUG]/[ABX], FIC, ΣFIC, the subsequent type of activity for every combination bacteria–natural compound–commercial antibiotic and MIC reduction (%) for each compound. Synergies are highlighted in pale grey and antibiotic reductions ≥ 50% are highlighted in dark grey.

Gram+/−	Bacteria	Compounds	MIC_comb_ (μg/mL)	Ratio [EUG]/[ABX] *	FIC	ΣFIC	Conclusion	MIC Reduction (%)
GRAM+	*L. monocytogenes*	EUG	250	714.3	0.25	0.50	Synergy	75
		GTM	3.1 *^a^*		0.25			75
		EUG	500	270.3	0.50	0.53	Addition	50
		AMP	3.9 *^a^*		0.03			97
		EUG	500	1111.1	0.50	0.51		50
		AMO	1.0 *^a^*		0.01			98
		EUG	500	44.4	0.50	1.00		50
		STM	100 *^a^*		0.50			50
		EUG	500	35.7	0.50	1.00		50
		ERY	62.5 *^a^*		0.50			50
		EUG	500	909.1	0.50	0.62		50
		TC	1.6 *^a^*		0.12			88
		EUG	500	196.1	0.50	1.00		50
		CHL	5 *^a^*		0.50			50
	*S. agalactiae*	EUG	500	34.1	0.50	1.00	Addition	50
		AMP	31.2 *^a^*		0.50			50
		EUG	500	35.7	0.50	1.00		50
		AMO	31.2 *^a^*		0.50			50
		EUG	500	1428.6	0.50	0.62		50
		GTM	3.3 *^a^*		0.12			88
		EUG	500	178.6	0.50	0.75		50
		STM	25 *^a^*		0.25			75
		EUG	500	35.7	0.50	1.00		50
		ERY	62.5 *^a^*		0.50			50
		EUG	500	158.7	0.50	0.62		50
		CHL	6.2 *^a^*		0.12			88
	*S. aureus*	EUG	250	714.3	0.25	0.37	Synergy	75
		GTM	3.1 *^a^*		0.12			88
		EUG	500	357.1	0.50	0.62	Addition	50
		STM	12.5 *^a^*		0.12			88
		EUG	125	29.3	0.12	1.12		88
		TC	12.5 *^a^*		1.00			0
		EUG	500	65.8	0.50	1.00		50
		CHL	15 *^a^*		0.50			50
GRAM−	*A. baumannii*	EUG	125	15.8	0.25	0.50	Synergy	75
		CHL	15.6 *^a^*		0.25			75
		EUG	250	8.5	0.50	1.00	Addition	50
		AMP	62.5 *^a^*		0.50			50
		EUG	250	8.9	0.50	1.50		50
		AMO	62.5 *^a^*		1.00			0
		EUG	250	181.8	0.50	1.00		50
		GTM	12.5 *^a^*		0.50			50
		EUG	250	112.4	0.50	1.50		50
		ERY	10 *^a^*		1.00			0
		EUG	250	7.4	0.50	4.50	Antagonism	50
		STM	300 *^a^*		4.00			−300
	*E. coli*	EUG	500	294.1	0.50	1.50	Addition	50
		GTM	15.6 *^a^*		1.00			0
		EUG	500	588.2	0.50	1.00		50
		STM	7.8 *^a^*		0.50			50
		EUG	250	17.9	0.50	1.50		50
		ERY	62.5 *^a^*		1.00			0
	*K. aerogenes*	EUG	250	0.7	0.50	1.00	Addition	50
		AMP	750 *^a^*		0.50			50
		EUG	250	0.6	0.50	1.50		50
		AMO	1000 *^a^*		1.00			0
		EUG	500	454.5	0.50	1.00		50
		ERY	5 *^a^*		0.50			−300
		EUG	250	3.9	0.50	4.50	Antagonism	50
		CHL	125 *^a^*		4.00			50
	*K. pneumoniae*	EUG	250	2.1	0.50	1.00	Addition	50
		AMP	250 *^a^*		0.50			50
		EUG	250	17.8	0.50	0.75		50
		AMO	31.2 *^a^*		0.25			75
		EUG	250	909.1	0.50	0.75		50
		STM	2.5 *^a^*		0.25			75
		EUG	250	71.4	0.50	0.75		50
		ERY	15.6 *^a^*		0.25			75
		EUG	250	256.4	0.50	0.62		50
		CHL	1.9 *^a^*		0.12			88
	*P. aerogenes*	EUG	250	4.3	0.50	1.50	Addition	50
		AMP	125 *^a^*		1.00			0
		EUG	250	1.1	0.5	1.50		50
		AMO	500 *^a^*		1.00			0
		EUG	250	142.9	0.50	1.50		50
		STM	15.6 *^a^*		1.00			0
		EUG	250	35.8	0.50	1.00		50
		ERY	31.2 *^a^*		0.50			50
		EUG	250	147.1	0.50	1.00		50
		TC	5 *^a^*		0.50			50
	*S. enterica*	EUG	250	178.6	0.25	0.50	Synergy	75
		STM	12.5 *^a^*		0.25			75
		EUG	500	71.4	0.50	1.50	Addition	50
		ERY	31.2 *^a^*		1.00			0
	*S. marcescens*	EUG	250	23.5	0.25	0.37	Synergy	75
		TC	31.2 *^a^*		0.12			88
		EUG	250	15.8	0.25	0.50		75
		CHL	31.2 *^a^*		0.25			75
		EUG	250	8.5	0.25	0.75	Addition	75
		AMP	62.5 *^a^*		0.50			50
		EUG	250	8.9	0.25	0.75		75
		AMO	62.5 *^a^*		0.50			50
		EUG	250	8.9	0.25	0.75		75
		ERY	125 *^a^*		0.50			50

* Calculated as MIC_comb_ EUG/MIC_comb_ ABX both in mol/L; *^a^* Ref. [2].

**Table 3:** Technical specifications of the bacterial strains and their growth conditions.

Bacterial Strains				
Gram	Name	T/°C	Broth	Provider
+	*Bacillus subtilis* (ATCC 6633)	30	BHI	Thermo Scientific
	*Enterococcus faecalis* (ATCC 19433)	37		
	*Listeria monocytogenes* (ATCC 7644)			
	*Streptococcus agalactiae* (ATCC 12386)			
	*Staphylococcus aureus* (ATCC 9144)		TSB	
−	*Acinetobacter baumannii* (ATCC 19606)			
	*Escherichia coli* (ATCC 25922)			
	*Klebsiella aerogenes* (ATCC 13048)	30	NB	
	*Klebsiella pneumoniae* (C6)	37		
	*Pasteurella aerogenes* (ATCC 27883)		BHI	
	*Proteus mirabilis* (ATCC 35659)		TSB	
	*Pseudomonas aeruginosa* (ATCC 27853)			
	*Salmonella enterica* (ATCC 13311)		NB	
	*Serratia marcescens* (ATCC 13880)	26		

BHI: Brain Heart Infusion; TSB: Trypticase Soy Broth; NB: Nutrient Broth.

## Data Availability

Data is contained within the article and Supplementary Materials.

## Supplementary Materials

The following supporting information can be downloaded at: https://www.mdpi.com/article/10.3390/ijms25137069/s1.
