# Supplementary material for: Bioactivity of Eugenol: A Potential Antibiotic Adjuvant with Minimal Ecotoxicological Impact
Source: Int J Mol Sci. 2024 Jun 27;25(13):7069. doi: 10.3390/ijms25137069 (PMC11241589; doi:10.3390/ijms25137069)
Supplement: Supplementary file 1 [file ijms-25-07069-s001.zip › ijms-3032688-supplementary.pdf]

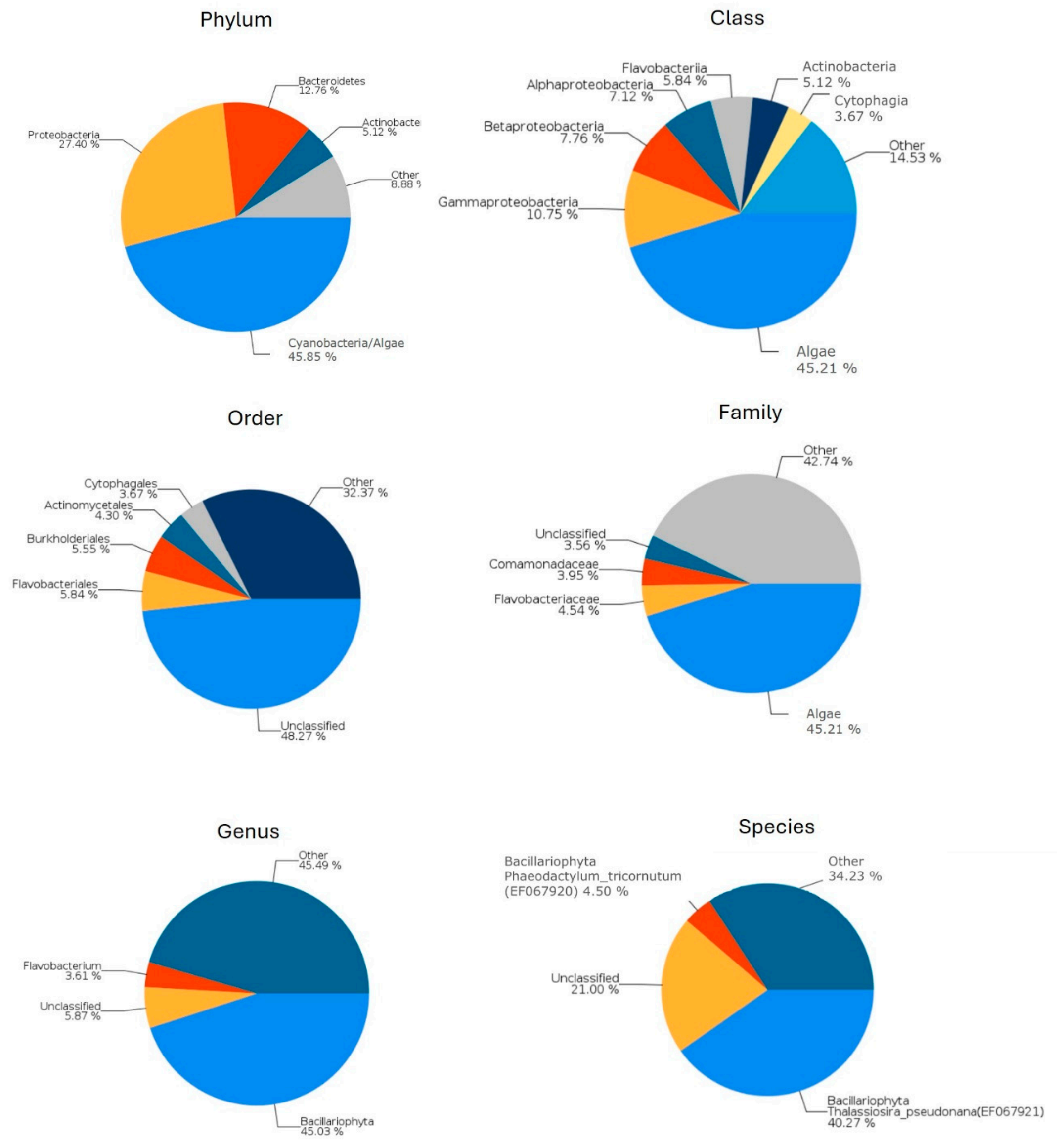


**Figure S1.** Main phylum, class, order, family, genus, and species classification results for water samples.

Notably, the most prevalent phyla were the primary producers Cyanobacteria/Algae (45.85%) and Proteobacteria (27.4%), with additional phyla including Bacteroidetes (12.76%) and Actinobacteria (5.12%) (Table S1). Proteobacteria exhibited three distinct classes: Alphaproteobacteria (7.12%), Betaproteobacteria (7.76%), and Gammaproteobacteria (10.75%), while 45.21% of the total reads belonged to Algae class. Other identified classes were Actinobacteria (5.12%) and Cytophagia (3.67%). The river water sample revealed bacteria from various orders, such as Aeromonadales (2.54%), Chromatiales (2.29%), Pseudomonadales (1.65%), and Alteromonadales (1.43%), all predominantly within the Gammaproteobacteria class (Table S1). Within Betaproteobacteria, Burkholderiales accounted for 5.55% of total reads, with Rhodocyclales and Neisseriales representing only 0.97% and 0.82%, respectively (Table S1). Additionally, Alphacianobacteria primarily comprised Rhodocacterales (3.32%), Sphingomonadales (1.19%), and Rhizobiales (1.32%) (Table S1). Cytophagales (3.67%), Actinomycetales (4.30%) and Flavobacteriales (5.84%) were other orders (not belonging to the cited classes), that must be highlighted. The most abundant families identified were the

primary producers Algae/Cyanobacteria (45.21%), Flavobacteriaceae (4.54%, Bacteroidetes phylum), and Comamonadaceae (3.95%, Proteobacteria phylum). At the genus level, Bacillariophyta emerged as the most abundant (45.03%), belonging to the Cyanobacteria phylum, all of them belonging to the species Thalassiosira (40.27%).

**Table S1.** Water sample complete taxonomy.

| Phylum | % | Class | % | Order | % | Family | % | Genus | % | Species | % |
|---|---|---|---|---|---|---|---|---|---|---|---|
| Cyanobacteria/Algae | 45.85 | Algae | 45.21 | Unclassified | 45.21 | *Algae* | 45.21 | *Bacillariophyta* | 45.03 | *Thalassiosira pseudonana* | 40.27 |
| | | | | | | | | | | *Phaeodactylum tricornutum* | 4.5 |
| | | | | | | | | | | Other | 0.26 |
| | | | | | | | | Other *Algae* | 0.18 | | |
| | | Other | 0.64 | | | | | | | | |
| Proteobacteria | 27.40 | Gammaproteobacteria | 10.75 | Aeromonadales | 2.54 | *Aeromonadaceae* | 2.52 | *Aeromonas* | 2.48 | *Unclassified* | 1.73 |
| | | | | | | | | | | *popofii* | 0.48 |
| | | | | | | | | | | Other | 0.18 |
| | | | | | | | | Other Aeromonadaceae | 0.03 | | |
| | | | | Chromatiales | 2.29 | *Chromatiaceae* | 2.04 | *Rheinheimera* | 1.90 | tilapiae | 0.77 |
| | | | | | | | | | | *chironomii* | 0.50 |
| | | | | | | | | | | *Mesophila* | 0.29 |
| | | | | | | | | | | Other | 0.34 |
| | | | | | | | | Other Chromatiaceae | 0.15 | | |
| | | | | | | *Ectothiorhodospiraceae* | 0.21 | *Natrocella* | 0.09 | *Woeseia oceani* | 0.08 |
| | | | | | | | | | | Other Natrocella | 0.08 |
| | | | | | | | | *Ectothiorhodospira* | 0.02 | *Ectothiorhodospira variabilis* | 0.02 |
| | | | | | | | | *Thioalkalivibrio* | 0.02 | Unclassified | 0.02 |
| | | | | | | | | | | *nitratireducens* | 0.0 |
| | | | | | | | | | | *versutus* | 0.0 |
| | | | | | | | | *Thiohalomonas* | 0.02 | *nitratireducens* | 0.02 |
| | | | | | | | | | | *denitrificans* | 0.0 |
| | | | | | | | | Unclassidied | 0.02 | | |
| | | | | | | | | *Acidiferrobacter* | 0.02 | Unclassified | 0.01 |
| | | | | | | | | | | *thiooxidans* | 0.01 |
| | | | | | | | | | | *Sulfuricaulis limicola* | 0.0 |
| | | | | | | | | | | *Sulfurifustis variabilis* | 0.0 |
| | | | | | | | | *Thiogranun* | 0.01 | *longum* | 0.01 |
| | | | | | | | | Other | 0.01 | | |
| | | | | Pseudomonadales | 1.65 | *Pseudomonadaceae* | 1.30 | *Cellvibrio* | 0.61 | fibriovorans | 0.27 |
| | | | | | | | | | | *gandavensis* | 0.14 |
| | | | | | | | | | | *fulvus* | 0.13 |

| | | | | | | | | | | | |
|---|---|---|---|---|---|---|---|---|---|---|---|
| | | | | | | | | | | Other | 0.06 |
| | | | | | | | | *Pseudomonas* | 0.55 | Unclassified | 0.18 |
| | | | | | | | | | | *peli* | 0.1 |
| | | | | | | | | | | *protegens* | 0.08 |
| | | | | | | | | | | *guguanensis* | 0.05 |
| | | | | | | | | | | Other | 0.13 |
| | | | | | | | | *Rhizobacter* | 0.12 | *fulvus* | 0.09 |
| | | | | | | | | | | *Rhizobacter sp.* | 0.01 |
| | | | | | | | | | | Other | 0.01 |
| | | | | | | | | Other | 0.03 | | |
| | | | | Alteromonadales | 1.43 | *Shewanellaceae* | 0.8 | *Shewanella* | 0.8 | Unclassified | 0.31 |
| | | | | | | | | | | *xiamenensis* | 0.19 |
| | | | | | | | | | | *putrefaciens* | 0.13 |
| | | | | | | | | | | *oneidensis* | 0.10 |
| | | | | | | | | | | Other | 0.07 |
| | | | | | | *Alteromonadaceae* | 0.59 | *Haliea* | 0.51 | *mediterranea* | 0.44 |
| | | | | | | | | | | *salexigens* | 0.04 |
| | | | | | | | | | | Other | 0.03 |
| | | | | | | | | Other Alteromonadaceae | 0.07 | | |
| | | | | Xanthomonadales | 1.06 | *Sinobacteraceae* | 0.59 | *Polibacter* | 0.54 | *uvarum* | 0.5 |
| | | | | | | | | | | Other | 0.04 |
| | | | | | | | | Other | 0.05 | | |
| | | | | | | *Xanthomonadaceae* | 0.47 | *Arenimonas* | 0.20 | *subflava* | 0.08 |
| | | | | | | | | | | *Aeromonas sp.* | 0.06 |
| | | | | | | | | | | *daechungensis* | 0.03 |
| | | | | | | | | | | Unclassified | 0.02 |
| | | | | | | | | | | Other | 0.01 |
| | | | | | | | | *Stenotrophomonas* | 0.06 | maltophilia | 0.04 |
| | | | | | | | | | | chelatiphaga | 0.02 |
| | | | | | | | | | | Other | 0.0 |
| | | | | | | | | *Thermononas* | 0.05 | *Thermomonas sp.* | 0.05 |
| | | | | | | | | *Lysobacter* | 0.05 | *brunescens* | 0.03 |
| | | | | | | | | | | *ginsengisoli* | 0.01 |
| | | | | | | | | | | *daejonensis* | 0.01 |
| | | | | | | | | | | Other | 0.01 |
| | | | | | | | | Other | 0.11 | | |
| | | Betaproteobacteria | 7.76 | Burkholderiales | 5.55 | *Comamonadaceae* | 3.95 | *Hydrogenophaga* | 1.98 | Unclassified | 1.17 |

| | | | | | | | | | | | |
|---|---|---|---|---|---|---|---|---|---|---|---|
| | | | | | | | | | | *taeniospiralis* | 0.43 |
| | | | | | | | | | | *caeni* | 0.30 |
| | | | | | | | | | | Other | 0.08 |
| | | | | | | | | *Limnohabitans* | 0.64 | *plantktonicus* | 0.39 |
| | | | | | | | | | | *parvus* | 0.14 |
| | | | | | | | | | | *australis* | 0.08 |
| | | | | | | | | | | Other | 0.04 |
| | | | | | | | | *Rhodoferax* | 0.5 | Unclassified | 0.25 |
| | | | | | | | | | | *ferrireducens* | 0.11 |
| | | | | | | | | | | *saidenbachensis* | 0.08 |
| | | | | | | | | | | *antarticus* | 0.05 |
| | | | | | | | | | | Other | 0.0 |
| | | | | | | | | Other | 0.82 | | |
| | | | | | | *Burkholderiales incertae sedis* | 0.62 | *Rubrivirax* | 0.13 | Other | 0.0 |
| | | | | | | | | | | *gelatinosus* | 0.12 |
| | | | | | | | | *Aquabacterium* | 0.13 | *commune* | 0.07 |
| | | | | | | | | | | *citratiphilum* | 0.03 |
| | | | | | | | | | | *fontiphilum* | 0.02 |
| | | | | | | | | | | Other | 0.02 |
| | | | | | | | | *Mitsuaria* | 0.08 | chitosanitabida | 0.08 |
| | | | | | | | | *Inhella* | 0.05 | *inkyongensis* | 0.05 |
| | | | | | | | | | | *fonticola* | 0.01 |
| | | | | | | | | *Paucibacter* | 0.05 | *toxinivorans* | 0.05 |
| | | | | | | | | *Leptothrix* | 0.05 | *discophora* | 0.04 |
| | | | | | | | | | | *mobilis* | 0.01 |
| | | | | | | | | *Sphaerotilus* | 0.04 | *montanus* | 0.02 |
| | | | | | | | | | | *natans* | 0.01 |
| | | | | | | | | | | Other | 0.0 |
| | | | | | | | | Other | 0.09 | | |
| | | | | | | Other | 0.98 | | | | |
| | | | | Rhodocyclales | 0.97 | *Rhodocyclaceae* | 0.97 | *Dechloromonas* | 0.35 | Unclassified | 0.33 |
| | | | | | | | | | | Other | 0.02 |
| | | | | | | | | Unclassified | 0.18 | | |
| | | | | | | | | *Sulfuritalea* | 0.07 | *hydrogenovorans* | 0.07 |
| | | | | | | | | *Sulfurisoma* | 0.05 | *sedimicola* | 0.05 |
| | | | | | | | | *Thaurea* | 0.06 | *aromatica* | 0.01 |
| | | | | | | | | | | Unclassified | 0.03 |

| | | | | | | | | | | | |
|---|---|---|---|---|---|---|---|---|---|---|---|
| | | | | | | | | | | *mechernichensis* | 0.01 |
| | | | | | | | | | | Other | 0.01 |
| | | | | | | | | Other | 0.27 | | |
| | | | | Neisseriales | 0.82 | *Neisseriaceae* | 0.82 | *Deefgea* | 0.22 | *chitinilytica* | 0.20 |
| | | | | | | | | | | *rivuli* | 0.02 |
| | | | | | | | | | | Other | 0.0 |
| | | | | | | | | *Chitinibacter* | 0.21 | *suncheonensis* | 0.19 |
| | | | | | | | | | | Other | 0.02 |
| | | | | | | | | *Vogesella* | 0.13 | Unclassified | 0.10 |
| | | | | | | | | | | *indigofera* | 0.02 |
| | | | | | | | | | | *oryzae* | 0.01 |
| | | | | | | | | | | Other | 0.0 |
| | | | | | | | | *Vitreoscilla* | 0.08 | *duriensis* | 0.06 |
| | | | | | | | | | | *filiformis* | 0.02 |
| | | | | | | | | *Chromobacterium* | 0.05 | *haemolyticum* | 0.02 |
| | | | | | | | | | | *subtsugae* | 0.01 |
| | | | | | | | | | | Unclassified | 0.01 |
| | | | | | | | | | | *aquaticum* | 0.01 |
| | | | | | | | | | | Other | 0.0 |
| | | | | | | | | *Formivibrio* | 0.03 | *citricus* | 0.03 |
| | | | | | | | | Other | 0.1 | | |
| | | Alphaproteobacteria | 7.12 | Rhodobacterales | 3.32 | *Rhodobacteraceae* | 3.32 | *Pseudorhodobacter* | 1.46 | *collinsensis* | 1.36 |
| | | | | | | | | | | Other | 0.1 |
| | | | | | | | | *Tabrizicola* | 0.41 | *aquatica* | 0.41 |
| | | | | | | | | *Gemnobacter* | 0.36 | *tilapiae* | 0.20 |
| | | | | | | | | | | Unclassified | 0.08 |
| | | | | | | | | | | *lanyuensis* | 0.05 |
| | | | | | | | | | | Other | 0.03 |
| | | | | | | | | *Citreicella* | 0.25 | Unclassified | 0.25 |
| | | | | | | | | *Rhodobacter* | 0.18 | *blasticus* | 0.14 |
| | | | | | | | | | | *azotoformans* | 0.02 |
| | | | | | | | | | | Other | 0.02 |
| | | | | Sphingomonadales | 1.19 | *Shingomonadaceae* | 0.99 | *Sphingorhabdus* | 0.64 | *wooponensis* | 0.53 |
| | | | | | | | | | | *Unclassified* | 0.07 |
| | | | | | | | | | | *Other* | 0.04 |
| | | | | | | | | *Shpingopyxis* | 0.11 | *Sphingoxyxis sp.* | 0.07 |
| | | | | | | | | | | Unclassified | 0.03 |

| | | | | | | | | | | | |
|---|---|---|---|---|---|---|---|---|---|---|---|
| | | | | | | | | | | Other | 0.01 |
| | | | | | | | | *Novosphingobium* | 0.10 | *Novosphingobium sp.* | 0.04 |
| | | | | | | | | | | *ginsenosidimutans* | 0.01 |
| | | | | | | | | | | *subterraneum* | 0.01 |
| | | | | | | | | | | Unclassified | 0.01 |
| | | | | | | | | | | Other | 0.03 |
| | | | | | | | | *Sphingomonas* | 0.07 | Unclassified | 0.04 |
| | | | | | | | | | | *fonticola* | 0.01 |
| | | | | | | | | | | Other | 0.02 |
| | | | | | | | | Other | 0.08 | | |
| | | | | | | *Erythrobacteraceae* | 0.20 | *Porphyrobacter* | 0.12 | sanguineus | 0.11 |
| | | | | | | | | | | Unclassified | 0.01 |
| | | | | | | | | | | Other | 0.01 |
| | | | | | | | | *Altererybacter* | 0.05 | *dongtanensis* | 0.03 |
| | | | | | | | | | | Unclassified | 0.01 |
| | | | | | | | | | | *indicus* | 0.01 |
| | | | | | | | | | | Other | 0.01 |
| | | | | | | | | *Erythrobacter* | 0.02 | Unclassified | 0.02 |
| | | | | | | | | | | *aquimaris* | 0.0 |
| | | | | | | Other | 0.0 | | | | |
| | | | | Rhizobiales | 1.32 | *Rhizobiaceae* | 0.37 | *Rhizobium* | 0.36 | Unclassified | 0.05 |
| | | | | | | | | | | *kunmingense* | 0.05 |
| | | | | | | | | | | *rosettiformans* | 0.03 |
| | | | | | | | | | | Other | 0.09 |
| | | | | | | | | Other | 0.01 | | |
| | | | | | | *Hyphomicrobiaceae* | 0.38 | *Hypomicrobium* | 0.12 | vulgare | 0.07 |
| | | | | | | | | | | Unclassified | 0.03 |
| | | | | | | | | | | sulfonivorans | 0.02 |
| | | | | | | | | | | Other | 0.0 |
| | | | | | | | | *Devosia* | 0.10 | Unclassified | 0.06 |
| | | | | | | | | | | *insulae* | 0.02 |
| | | | | | | | | | | Other | 0.01 |
| | | | | | | | | *Filomicrobium* | 0.04 | *insigne* | 0.04 |
| | | | | | | | | | | Other | 0.0 |
| | | | | | | | | *Pedomicrobium* | 0.03 | Unclassified | 0.02 |
| | | | | | | | | | | *manganicum* | 0.01 |
| | | | | | | | | Other | 0.08 | | |

| | | | | | | | | | | | |
|---|---|---|---|---|---|---|---|---|---|---|---|
| | | | | | | *Rhodobiaceae* | 0.21 | *Rhodoviaceae* | 0.16 | *oligomobilis* | 0.14 |
| | | | | | | | | | | *appendicifer* | 0.02 |
| | | | | | | | | | | Other | 0.02 |
| | | | | | | | | *Anderseniella* | 0.01 | *baltica* | 0.01 |
| | | | | | | | | Unclassified | 0.02 | | |
| | | | | | | | | *Parvibaculum* | 0.01 | *marinus* | 0.01 |
| | | | | | | | | Other | 0.0 | | |
| | | | | | | *Rhizobiales incertae sedis* | 0.10 | *Alsobacter* | 0.05 | *metallidurans* | 0.05 |
| | | | | | | | | *Phreatobacter* | 0.03 | *Alpha proteobacterium* | 0.03 |
| | | | | | | | | *Vasilyevaea* | 0.01 | Unclassified | 0.0 |
| | | | | | | | | | | *mishustinii* | 0.0 |
| | | | | | | | | Other | 0.01 | | |
| | | | | | | *Phyllobacteriaceae* | 0.09 | *Aquamicrobium* | 0.03 | *defluvii* | 0.03 |
| | | | | | | | | | | Unclassified | 0.0 |
| | | | | | | | | *Nitratireductor* | 0.01 | *soli* | 0.01 |
| | | | | | | | | *Hoeflea* | 0.02 | *marina* | 0.01 |
| | | | | | | | | | | *halophila* | 0.0 |
| | | | | | | | | | | *aestuarii* | 0.0 |
| | | | | | | | | | | *cellulosilytica* | 0.0 |
| | | | | | | | | Unclassified | 0.02 | | |
| | | | | | | | | Phillobacterium | 0.0 | Unclassified | 0.0 |
| | | | | | | | | Other | 0.0 | | |
| | | | | | | Other | 0.18 | | | | |
| | | | | Rhodospirillales | 0.64 | *Rhodospirillaceae* | 0.49 | *Magnetovibrio* | 0.12 | *blakemorei* | 0.12 |
| | | | | | | | | *Azospirillum* | 0.10 | *irakense* | 0.07 |
| | | | | | | | | | | Unclassified | 0.02 |
| | | | | | | | | | | *thiophillum* | 0.01 |
| | | | | | | | | | | azospirillum | 0.01 |
| | | | | | | | | *Novispirillum* | 0.08 | *Unclassified* | 0.07 |
| | | | | | | | | | | *Other* | 0.01 |
| | | | | | | | | *Pelagibius* | 0.03 | *litoralis* | 0.03 |
| | | | | | | | | | | Other | 0.0 |
| | | | | | | | | *Aestuariispira* | 0.03 | Rhodospirillaceae bacterium | 0.03 |
| | | | | | | | | Other | 0.13 | | |
| | | | | | | *Acetobacteriaceae* | 0.11 | *Roseomonas* | 0.09 | *Unclassified* | 0.03 |
| | | | | | | | | | | *lacus* | 0.02 |

| | | | | | | | | | | | |
|---|---|---|---|---|---|---|---|---|---|---|---|
| | | | | | | | | | | *riguiloci* | 0.02 |
| | | | | | | | | | | Other | 0.02 |
| | | | | | | | | *Stella* | 0.01 | *vacuolata* | 0.01 |
| | | | | | | | | | | Unclassified | 0.00 |
| | | | | | | | | *Nguyenibacter* | 0.01 | *intestini* | 0.01 |
| | | | | | | | | Other | 0.01 | | |
| | | | | Other | 0.65 | | | | | | |
| | | Other Proteobacteria | 1.17 | | | | | | | | |
| Bacteroidetes | 12.76 | Flavobacteriia | 5.84 | Flavobacteriales | 5.84 | *Flavobactariaceae* | 4.54 | *Flavobacterium* | 3.61 | Unclassified | 2.16 |
| | | | | | | | | | | Other | 1.45 |
| | | | | | | | | *Polaribacter* | 0.54 | *gangjinensis* | 0.54 |
| | | | | | | | | Other | 0.38 | | |
| | | | | | | *Cryomorphaceae* | 1.30 | *Brumimicrobium* | 0.66 | *mesophilum* | 0.61 |
| | | | | | | | | | | Other | 0.05 |
| | | | | | | | | *Fluviicola* | 0.47 | *taffensis* | 0.45 |
| | | | | | | | | | | Other | 0.01 |
| | | | | | | | | *Salinirepens* | 0.07 | *amaniensis* | 0.07 |
| | | | | | | | | Other | 0.11 | | |
| | | | | | | Other | 0.0 | | | | |
| | | Cytophagia | 3.67 | Cytophagales | 3.67 | *Cytophagaceae* | 2.97 | *Arcicella* | 1.82 | Unclassified | 1.81 |
| | | | | | | | | | | Other | 0.01 |
| | | | | | | | | *Fluviimonas* | 0.54 | *pallidilutea* | 0.54 |
| | | | | | | | | *Pseudarcicella* | 0.22 | *Aceicella sp.* | 0.22 |
| | | | | | | | | Other | 0.39 | | |
| | | | | | | Unclassified | 0.32 | *Ohtaekwangia* | 0.10 | *koreensis* | 0.09 |
| | | | | | | | | | | Other | 0.0 |
| | | | | | | | | *Unclassified* | 0.16 | | |
| | | | | | | | | Chryseolinea | 0.07 | Sphingobacteria bacterium | 0.07 |
| | | | | | | *Cyclobacteriaceae* | 0.22 | *Algoriphagus* | 0.18 | *shivajiensis* | 0.08 |
| | | | | | | | | | | *aquaductus* | 0.04 |
| | | | | | | | | | | Unclassified | 0.04 |
| | | | | | | | | | | Other | 0.02 |
| | | | | | | | | *Fontibacter* | 0.01 | *Unclassified* | 0.01 |
| | | | | | | | | Other | 0.02 | | |
| | | | | | | Other | 0.15 | | | | |
| | | Shingobacteriia | 1.95 | Shingobacteriales | 1.95 | *Saprospiraceae* | 0.86 | *Haliscomenobacter* | 0.31 | *hydrossis* | 0.31 |
| | | | | | | | | *Lewinella* | 0.24 | Unclassified | 0.21 |

| | | | | | | | | | | | |
|---|---|---|---|---|---|---|---|---|---|---|---|
| | | | | | | | | | | Other | 0.03 |
| | | | | | | | | *Portibacter* | 0.18 | *lacus* | 0.18 |
| | | | | | | | | *Phaeodactylibacter* | 0.06 | Unclassified | 0.05 |
| | | | | | | | | | | Other | 0.0 |
| | | | | | | | | Other | 0.01 | | |
| | | | | | | *Chitinophagaceae* | 0.86 | *Sediminibacterium* | 0.33 | *salmoneum* | 0.21 |
| | | | | | | | | | | Unclassified | 0.07 |
| | | | | | | | | | | *Sediminibacterium sp.* | 0.04 |
| | | | | | | | | | | Other | 0.01 |
| | | | | | | | | Unclassified | 0.27 | | |
| | | | | | | | | *Chitinophaga* | 0.07 | *sancti* | 0.04 |
| | | | | | | | | | | Unclassified | 0.01 |
| | | | | | | | | | | *jiangningensis* | 0.01 |
| | | | | | | | | | | Other | 0.01 |
| | | | | | | | | *Parasegetibacter* | 0.05 | *Parasegetibacter sp.* | 0.05 |
| | | | | | | | | Other | 0.13 | | |
| | | | | | | *Shingobacteriaceae* | 0.22 | *Pedobacter* | 0.10 | *terricola* | 0.03 |
| | | | | | | | | | | *alpinus* | 0.02 |
| | | | | | | | | | | Unclassified | 0.02 |
| | | | | | | | | | | *glucosidilyticus* | 0.01 |
| | | | | | | | | | | Other | 0.02 |
| | | | | | | | | *Solitalea* | 0.04 | *canadensis* | 0.04 |
| | | | | | | | | | | Unclassified | 0.01 |
| | | | | | | | | *Mucilaginibacter* | 0.03 | *sabulilitoris* | 0.01 |
| | | | | | | | | | | *litoreus* | 0.01 |
| | | | | | | | | | | Unclassified | 0.0 |
| | | | | | | | | | | *Mucilaginibacter sp.* | 0.0 |
| | | | | | | | | | | Other | 0.0 |
| | | | | | | | | Unclassified | 0.03 | | |
| | | | | | | | | *Parapedobacter* | 0.01 | *soli* | 0.01 |
| | | | | | | | | Other | 0.0 | | |
| | | | | | | Other | 0.01 | | | | |
| | | Bacteroidia | 1.03 | Bacteroidales | 1.03 | *Bacteroidaceae* | 0.34 | *Bacteroides* | 0.34 | *plebeius* | 0.12 |
| | | | | | | | | | | *coprocola* | 0.08 |
| | | | | | | | | | | *caecigallinarum* | 0.06 |
| | | | | | | | | | | Other | 0.08 |
| | | | | | | | | Other | 0.0 | | |

| | | | | | | | | | | | |
|---|---|---|---|---|---|---|---|---|---|---|---|
| | | | | | | *Prolixibacteraceae* | 0.19 | *Mariniphaga* | 0.14 | *sediminis* | 0.09 |
| | | | | | | | | | | Unclassified | 0.03 |
| | | | | | | | | | | *Roseimarinus sediminis* | 0.02 |
| | | | | | | | | | | Other | 0.0 |
| | | | | | | | | Unclassified | 0.05 | | |
| | | | | | | | | Other | 0.01 | | |
| | | | | | | *Porphyromonadaceae* | 0.20 | *Barnesiella* | 0.05 | *intestinale* | 0.04 |
| | | | | | | | | | | *intestinihominis* | 0.01 |
| | | | | | | | | *Dysgonomonas* | 0.05 | *alginatilytica* | 0.03 |
| | | | | | | | | | | Unclassified | 0.02 |
| | | | | | | | | | | Other | 0.01 |
| | | | | | | | | *Paludibacter* | 0.02 | *propionicigenes* | 0.02 |
| | | | | | | | | *Parabacterioides* | 0.02 | *johnsonii* | 0.01 |
| | | | | | | | | | | *gordonii* | 0.01 |
| | | | | | | | | | | *merdae* | 0.0 |
| | | | | | | | | | | Other | 0.0 |
| | | | | | | | | *Acetobacteroides* | 0.01 | *Acetobacteroides bacterium* | 0.01 |
| | | | | | | | | *Petrimonas* | 0.01 | *sulfuriphila* | 0.01 |
| | | | | | | | | *Butyricimonas* | 0.01 | *massiliensis* | 0.01 |
| | | | | | | | | Other | 0.02 | | |
| | | | | | | *Rikenellaceae* | 0.13 | *Alistipes* | 0.09 | Unclassified | 0.03 |
| | | | | | | | | | | *indistinctus* | 0.02 |
| | | | | | | | | | | *senegalensis* | 0.02 |
| | | | | | | | | | | *ihumii* | 0.01 |
| | | | | | | | | | | Other | 0.01 |
| | | | | | | | | *Mucinivorans* | 0.04 | *hirudinis* | 0.04 |
| | | | | | | | | Other | 0.01 | | |
| | | | | | | Other | 0.16 | | | | |
| | | Other | 0.27 | | | | | | | | |
| Actinobacteria | 5.12 | Actinobacteria | 5.12 | Actinomycetales | 4.30 | | | | | | |
| | | | | Other | 0.82 | | | | | | |
| Other | 13.32 | | | | | | | | | | |

**Table S2.** AWCD of water and soil samples for the 5 groups of metabolites as a function of time (h) for the control group and the different doses of eugenol.

| | WATER | | | | | SOIL | | | | |
|---|---|---|---|---|---|---|---|---|---|---|
| | [Eugenol] (mg/L) | | | | | | | | | |
| **T (h)** | **0** | **0.1** | **10** | **100** | **1000** | **0** | **0.1** | **10** | **100** | **1000** |
| | **Polymers** | | | | | | | | | |
| **0** | 0.05 | 0.04 | 0.05 | 0.06 | 0.03 | 0.00 | 0.01 | 0.00 | 0.01 | 0.00 |
| | | | | | | | | | | |
| **24** | 0.05 | 0.04 | 0.11 | 0.05 | 0.04 | 0.05 | 0.05 | 0.05 | 0.04 | 0.00 |
| **48** | 0.26 | 0.34 | 0.34 | 0.19 | 0.03 | 0.69 | 0.78 | 0.70 | 0.67 | 0.06 |
| **72** | 0.54 | 0.67 | 0.64 | 0.42 | 0.07 | 1.29 | 1.28 | 1.15 | 1.10 | 0.07 |
| **96** | 0.83 | 0.94 | 0.84 | 0.62 | 0.08 | 1.78 | 1.78 | 1.76 | 1.51 | 0.07 |
| **120** | 1.12 | 1.14 | 1.03 | 0.82 | 0.11 | 2.09 | 2.02 | 2.18 | 1.70 | 0.09 |
| **144** | 1.25 | 1.28 | 1.13 | 0.94 | 0.16 | 2.18 | 2.05 | 2.30 | 1.78 | 0.09 |
| **168** | 1.32 | 1.37 | 1.20 | 1.00 | 0.16 | 2.23 | 2.04 | 2.32 | 1.90 | 0.10 |
| | **Carbohydrates** | | | | | | | | | |
| **0** | 0.00 | 0.00 | 0.01 | 0.01 | 0.01 | 0.01 | 0.01 | 0.01 | 0.01 | 0.01 |
| **24** | 0.03 | 0.01 | 0.06 | 0.01 | 0.02 | 0.04 | 0.03 | 0.02 | 0.02 | 0.01 |
| **48** | 0.46 | 0.35 | 0.53 | 0.32 | 0.02 | 0.76 | 0.88 | 0.85 | 0.58 | 0.01 |
| **72** | 0.75 | 0.62 | 0.83 | 0.64 | 0.01 | 1.29 | 1.31 | 1.33 | 0.90 | 0.00 |
| **96** | 0.94 | 0.79 | 0.98 | 0.88 | 0.01 | 1.48 | 1.37 | 1.56 | 1.08 | 0.01 |
| **120** | 1.04 | 0.88 | 1.10 | 1.02 | 0.01 | 1.71 | 1.44 | 1.57 | 1.16 | 0.01 |
| **144** | 1.12 | 0.93 | 1.13 | 1.06 | 0.02 | 1.75 | 1.46 | 1.61 | 1.18 | 0.01 |
| **168** | 1.16 | 0.95 | 1.15 | 1.06 | 0.03 | 1.82 | 1.51 | 1.62 | 1.20 | 0.01 |
| | **Carboxylic and acetic acids** | | | | | | | | | |
| **0** | 0.00 | 0.00 | 0.00 | 0.00 | 0.00 | 0.00 | 0.00 | 0.00 | 0.00 | 0.01 |
| **24** | 0.02 | 0.01 | 0.03 | 0.01 | 0.02 | 0.04 | 0.03 | 0.03 | 0.01 | 0.01 |
| **48** | 0.16 | 0.18 | 0.22 | 0.13 | 0.02 | 0.62 | 0.66 | 0.61 | 0.60 | 0.01 |
| **72** | 0.43 | 0.53 | 0.51 | 0.42 | 0.02 | 1.08 | 1.06 | 0.94 | 0.96 | 0.00 |
| **96** | 0.56 | 0.68 | 0.69 | 0.56 | 0.02 | 1.31 | 1.21 | 1.21 | 1.14 | 0.01 |
| **120** | 0.70 | 0.77 | 0.78 | 0.66 | 0.03 | 1.44 | 1.25 | 1.32 | 1.24 | 0.01 |
| **144** | 0.80 | 0.83 | 0.84 | 0.71 | 0.04 | 1.45 | 1.26 | 1.34 | 1.26 | 0.02 |
| **168** | 0.83 | 0.90 | 0.89 | 0.75 | 0.05 | 1.50 | 1.27 | 1.31 | 1.26 | 0.02 |
| | **Amino acids** | | | | | | | | | |
| **0** | 0.00 | 0.00 | 0.00 | 0.00 | 0.01 | 0.00 | 0.00 | 0.00 | 0.00 | 0.00 |
| **24** | 0.02 | 0.00 | 0.03 | 0.00 | 0.01 | 0.04 | 0.04 | 0.03 | 0.02 | 0.00 |
| **48** | 0.19 | 0.16 | 0.20 | 0.06 | 0.02 | 0.50 | 0.49 | 0.56 | 0.47 | 0.00 |
| **72** | 0.48 | 0.40 | 0.38 | 0.23 | 0.02 | 0.87 | 0.80 | 0.98 | 0.88 | 0.00 |
| **96** | 0.69 | 0.64 | 0.55 | 0.38 | 0.02 | 1.14 | 1.09 | 1.26 | 1.20 | 0.00 |
| **120** | 0.90 | 0.89 | 0.76 | 0.55 | 0.05 | 1.37 | 1.20 | 1.32 | 1.30 | 0.02 |
| **144** | 1.05 | 1.07 | 0.89 | 0.62 | 0.07 | 1.44 | 1.31 | 1.43 | 1.32 | 0.05 |
| **168** | 1.13 | 1.22 | 0.97 | 0.70 | 0.09 | 1.61 | 1.44 | 1.51 | 1.34 | 0.09 |
| | **Amines/amides** | | | | | | | | | |
| **0** | 0.00 | 0.01 | 0.00 | 0.00 | 0.00 | 0.01 | 0.01 | 0.01 | 0.01 | 0.01 |
| **24** | 0.01 | 0.00 | 0.02 | 0.00 | 0.04 | 0.03 | 0.03 | 0.03 | 0.01 | 0.01 |
| **48** | 0.05 | 0.07 | 0.12 | 0.03 | 0.04 | 1.05 | 0.76 | 0.82 | 0.56 | 0.01 |
| **72** | 0.36 | 0.33 | 0.30 | 0.30 | 0.03 | 1.55 | 1.16 | 1.33 | 1.09 | 0.00 |
| **96** | 0.65 | 0.47 | 0.41 | 0.61 | 0.04 | 1.72 | 1.38 | 1.56 | 1.37 | 0.00 |
| **120** | 0.83 | 0.56 | 0.47 | 0.67 | 0.05 | 1.89 | 1.43 | 1.60 | 1.50 | 0.00 |
| **144** | 0.91 | 0.63 | 0.47 | 0.70 | 0.06 | 1.80 | 1.38 | 1.58 | 1.54 | 0.00 |
| **168** | 0.92 | 0.72 | 0.47 | 0.68 | 0.07 | 1.72 | 1.35 | 1.53 | 1.53 | 0.00 |

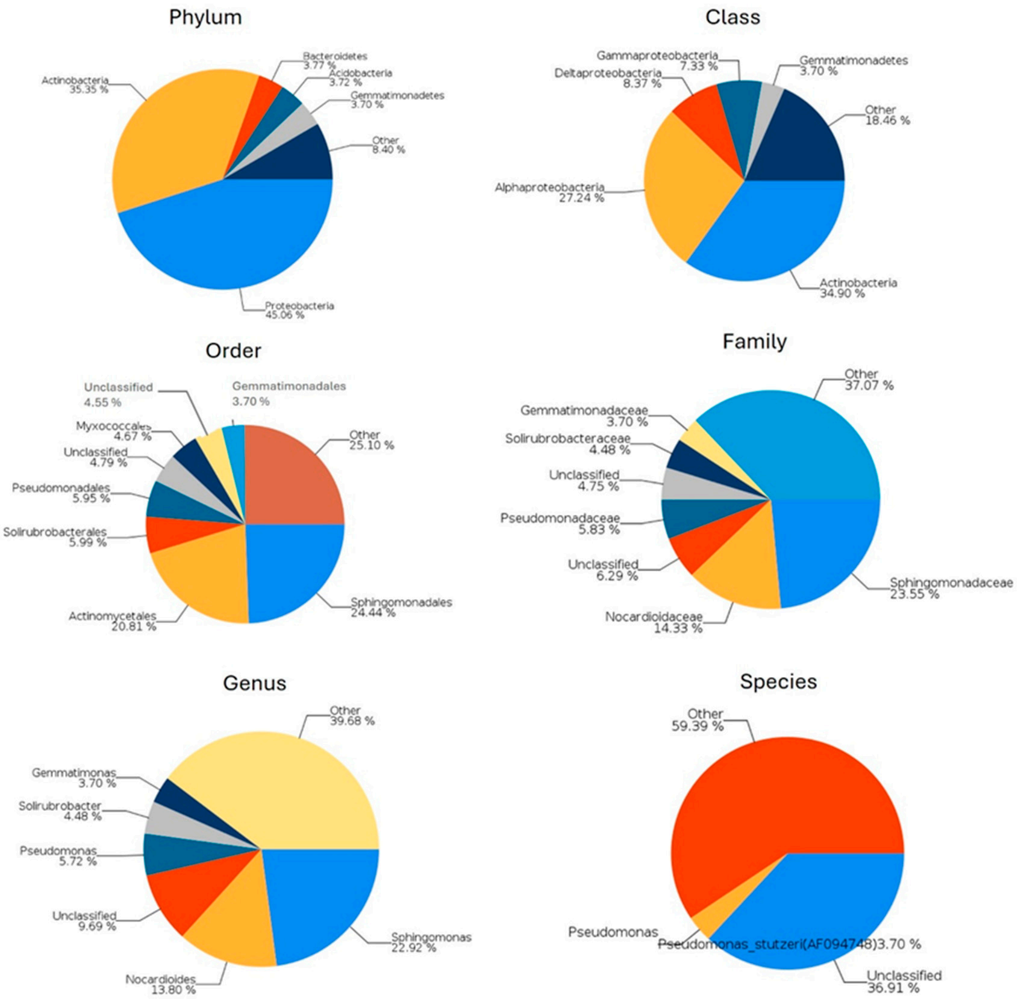


**Figure S2.** Main phylum, class, order, family, genus, and species classification results for soil samples.

Figure S2 (and Table S3) illustrates the prevalence of taxa across various taxonomic levels within the soil samples. Predominantly, the phyla Actinobacteria (35.35%) and Proteobacteria (45.06%) were observed, with a lesser presence of Bacteroidetes (3.77%), Acidobacteria (3.72%) and Gemmatimonadetes (3.70%). Within Actinobacteria, only the class Actinobacteria (34.90%) was identified, while Proteobacteria exhibited three prominent classes: Alphaproteobacteria (27.24%), Deltaproteobacteria (8.37%) and Gammaproteobacteria (7.33%). Notably, diverse orders were detected within the class Actinobacteria, such as Actinomycetales (20.81%), Solirubrobacterales (5.99%), Acidimicrobiales (2.89%), Gaillales (2.07%) (Table S3). In the Alphaproteobacteria class, Sphingomonadales accounted for 24.44% of total reads (Table S3). Within Deltaproteobacteria and Gammaproteobacteria classes, Myxococcales (4.67%) and Pseudomonadales (5.95%) were the most abundant orders, respectively (Table S3). The most prevalent families identified were Sphingomonadaceae (23.55%, Proteobateria phylum) and Nocardioidaceae (14.33%, Actinobacteria phylum). At the genus level, Nocardiodes (13.88%) and Sphingomonas (22.92%) emerged as the most abundant representatives within the Actinobacteria and Proteobacteria phyla, respectively. Table S3 provides more specific information about the taxonomic classification of soil microorganisms.

**Table S3.** Soil sample complete taxonomy.

| Phylum | % | Class | % | Order | % | Family | % | Genus | % | Species | % |
|---|---|---|---|---|---|---|---|---|---|---|---|
| Proteobacteria | 45.06 | Alphaproteobacteria | 27.24 | Sphingomonadales | 24.44 | *Sphingomonadaceae* | 23.55 | *Sphingomonas* | 22.92 | Unclassified | 10.38 |
| | | | | | | | | | | Other | 12.54 |
| | | | | | | | | *Other* | 0.63 | | |
| | | | | | | Other | 0.89 | | | | |
| | | | | Other | 2.79 | | | | | | |
| | | Deltaproteobacteria | 8.37 | Myxococcales | 4.67 | *Vulgatibacteraceae* | 3.42 | *Vulgatibacter* | 3.42 | *incomptus* | 3.42 |
| | | | | | | *Myxococcaceae* | 0.44 | *Aggregicoccus* | 0.37 | *edonensis* | 0.37 |
| | | | | | | | | *Angiococcus* | 0.04 | Unclassified | 0.04 |
| | | | | | | | | Other | 0.02 | | |
| | | | | | | *Cystobacteraceae* | 0.44 | *Anaeromyxobacter* | 0.25 | *dehalogenans* | 0.25 |
| | | | | | | | | *Stigmatella* | 0.16 | Unclassified | 0.16 |
| | | | | | | | | *Cystobacter* | 0.03 | *gracillis* | 0.02 |
| | | | | | | | | | | *violaceus* | 0.01 |
| | | | | | | | | | | *gephyra* | 0.0 |
| | | | | | | | | | | Other | 0.0 |
| | | | | | | | | Other | 0.0 | | |
| | | | | Desulfuromonadales | 1.92 | *Geobacteraceae* | 1.86 | Geobacter | 1.86 | Unclassified | 1.85 |
| | | | | | | | | | | Other | 0.01 |
| | | | | | | | | Other | 0.01 | | |
| | | | | | | Other | 0.06 | | | | |
| | | | | Bdellovibrionales | 0.72 | *Bacteriovoracaceae* | 0.67 | Peredibacter | 0.67 | Starrii | 0.67 |
| | | | | | | | | Other | 0.0 | | |
| | | | | | | *Bdellovibrionaceae* | 0.05 | *Bdellovibrio* | 0.05 | *exovorus* | 0.04 |
| | | | | | | | | | | *bacteriovorus* | 0.01 |
| | | | | | | | | | | Other | 0.0 |
| | | | | Desulfovibrionales | 0.56 | *Desulfovibrionaceae* | 0.54 | *Desulfovibrio* | 0.54 | Unclassified | 0.53 |
| | | | | | | | | | | Other | 0.0 |
| | | | | | | | | Other | 0.0 | | |
| | | | | | | Other | 0.02 | | | | |
| | | | | Other | 0.5 | | | | | | |
| | | Ganmaproteobacteri a | 7.33 | Pseudomonadales | 5.95 | *Pseudomonadaceae* | 5.83 | *Pseudomonas* | 5.72 | *stutzeri* | 3.70 |
| | | | | | | | | | | *pseudoalcaligenes* | 0.96 |
| | | | | | | | | | | Unclassified | 0.81 |
| | | | | | | | | | | Other | 0.26 |

| | | | | | | | | | | | |
|---|---|---|---|---|---|---|---|---|---|---|---|
| | | | | | | | | Other | 0.11 | | |
| | | | | | | Other | 0.12 | | | | |
| | | | | Xanthomonadales | 0.54 | *Xanthomonadaceae* | 0.53 | *Stenotrophomonas* | 0.38 | *maltophilia* | 0.26 |
| | | | | | | | | | | Unclassified | 0.08 |
| | | | | | | | | | | *rhizophila* | 0.04 |
| | | | | | | | | | | Other | 0.01 |
| | | | | | | | | *Luteimonas* | 0.05 | *Lysobacter panaciterrae* | 0.05 |
| | | | | | | | | | | Other | 0.0 |
| | | | | | | | | *Lysobacter* | 0.05 | Unclassified | 0.02 |
| | | | | | | | | | | *lycopersici* | 0.01 |
| | | | | | | | | | | *dokdonensis* | 0.01 |
| | | | | | | | | | | *bugurensis* | 0.01 |
| | | | | | | | | | | *soli* | 0.01 |
| | | | | | | | | | | Other | 0.01 |
| | | | | | | | | Other | 0.05 | | |
| | | | | | | Other | 0.01 | | | | |
| | | | | Other | 0.84 | | | | | | |
| | | Other | 2.12 | | | | | | | | |
| Actinobacteria | 35.35 | Actinobacteria | 34.90 | Actinomycetales | 20.81 | *Nocardioidaceae* | 14.33 | *Nocardioides* | 13.80 | Unclassified | 3.34 |
| | | | | | | | | | | *islandensis* | 3.25 |
| | | | | | | | | | | *tritolerans* | 1.26 |
| | | | | | | | | | | *mesophilus* | 1.26 |
| | | | | | | | | | | *aestuarii* | 1.23 |
| | | | | | | | | | | Other | 3.45 |
| | | | | | | | | Other | 0.53 | | |
| | | | | | | | | | | | |
| | | | | | | *Micrococcaceae* | 0.94 | *Arthrobacter* | 0.90 | Unclassified | 0.78 |
| | | | | | | | | | | Other | 0.12 |
| | | | | | | | | *Other* | *0.04* | | |
| | | | | | | *Geodermatophilaceae* | 1.31 | *Modestobacter* | 0.60 | *lapidis* | *0.36* |
| | | | | | | | | | | Unclassified | 0.22 |
| | | | | | | | | | | Other | 0.02 |
| | | | | | | | | *Geodermatophilus* | *0.46* | *siccatus* | *0.35* |
| | | | | | | | | | | *nigrescens* | *0.04* |
| | | | | | | | | | | Other | *0.07* |
| | | | | | | | | *Blastococcus* | *0.22* | *agregatus* | *0.12* |

| | | | | | | | | | | | |
|---|---|---|---|---|---|---|---|---|---|---|---|
| | | | | | | | | | | *jejuensis* | *0.06* |
| | | | | | | | | | | *endophyticus* | *0.03* |
| | | | | | | | | | | Other | *0.01* |
| | | | | | | | | *Other* | *0.03* | | |
| | | | | | | Other | 4.23 | | | | |
| | | | | Solirubrobacterales | 5.99 | *Solirubrobacteraceae* | 4.48 | *Solirubrobacter* | 4.48 | *gingenosidimutans* | 2.0 |
| | | | | | | | | | | *Solirubrobacter sp.* | 1.95 |
| | | | | | | | | | | Unclassified | 0.49 |
| | | | | | | | | | | *Other* | 0.04 |
| | | | | | | *Conexibacteraceae* | 1.18 | *Conexibacter* | 1.18 | Unclassified | 0.81 |
| | | | | | | | | | | *arvalis* | 0.28 |
| | | | | | | | | | | Other | 0.09 |
| | | | | | | Other | 0.33 | | | | |
| | | | | Acidimicrobiales | 2.89 | *Acidimicrobineae incertae sedis* | 1.39 | *Aciditerrimonas* | 1.39 | *ferrireducens* | 1.39 |
| | | | | | | *Lamiaceae* | 0.75 | *Aquihabitans* | 0.66 | *Aciditerrimonas sp.* | 0.66 |
| | | | | | | | | *Lamia* | 0.08 | majanohamensis | 0.08 |
| | | | | | | | | Other | 0.0 | | |
| | | | | | | *Acidimicrobiaceae* | 0.67 | *Ilumatobacter* | 0.53 | *fluminis* | 0.51 |
| | | | | | | | | | | Other | 0.02 |
| | | | | | | | | *Ferrithrix* | 0.13 | *thermotolerans* | 0.13 |
| | | | | | | | | Other | 0.01 | | |
| | | | | | | Other | 0.08 | | | | |
| | | | | Gaiellales | 2.07 | *Gaiellaceae* | 2.07 | *Gaiella* | 2.07 | *occulta* | 2.07 |
| | | | | Other | 3.15 | | | | | | |
| | | Other | 0.45 | | | | | | | | |
| Other bacteria | 19.06 | | | | | | | | | | |

**Table S4.** Physico-chemical parameters of water and soil samples.

| **River** | | **Soil** | |
|---|---|---|---|
| Parameter | Value | Parameter | Value |
| Conductivity (mS/cm) | 2,320.0 | Clay content (%) | 12.3 |
| Total suspended solids (mg/L) | 5.0 | Sand content (%) | 51.5 |
| Organic matter (mg/L) | 3.0 | Silt content (%) | 37.1 |
| Total dissolved solids(mg/L) | 1,590.1 | K (mg/L) | 110.2 |
| Carbonates (mg/L) | 0.0 | P Olsen (mg/Kg) | 10.2 |
| Bicarbonates (mg/L) | 248.9 | Ca meq 100 | 23.6 |
| Fluorides (mg/L) | 0.09 | $EC_{1:5}$ (dS/m) | 0.6 |
| Chlorides (mg/L) | 477.0 | Total nitrogen (%) | 0.1 |
| Nitrites (mg/L) | 0.0 | $CaCO_3$ (%) | 42.5 |
| Bromides (mg/L) | 0.5 | pH | 8.1 |
| Nitrates (mg/L) | 14.6 | | |
| Phosphates (mg/L) | 0.0 | | |
| Sulphates (mg/L) | 356.5 | | |
| Total alkalinity (mg/L) | 287.4 | | |
| Total organic carbon (mg/L) | 2.89 | | |
| Total nitrogen (mg/L) | 3.8 | | |
| pH | 7.8 | | |
